# AKT Inhibitor SC66 Inhibits Proliferation and Induces Apoptosis in Human Glioblastoma Through Down-Regulating AKT/β-Catenin Pathway

**DOI:** 10.3389/fphar.2020.01102

**Published:** 2020-07-31

**Authors:** Lun Gao, Junhui Liu, Pengfei Xu, Gang Deng, Baohui Liu, Fanen Yuan, Yinqiu Tan, Qian Sun, Yang Xu, Huikai Zhang, Yangzhi Qi, Shoumeng Han, Kun Yang, Rongxin Geng, Hongxiang Jiang, Qianxue Chen

**Affiliations:** ^1^ Department of Neurosurgery, Renmin Hospital of Wuhan University, Wuhan, China; ^2^ Central Laboratory, Renmin Hospital of Wuhan University, Wuhan, China; ^3^ Center for Science Research, The 7th Affiliated Hospital of Sun Yat-Sen University, Shenzhen, China

**Keywords:** SC66, glioblastoma multiforme, epithelial-to-mesenchymal transition, proliferation, apoptosis, AKT/β-catenin pathway

## Abstract

Glioblastoma multiforme (GBM) is the most common intracranial malignancy in adults with the highest degree of malignancy and mortality. Due to its nature of diffuse invasiveness and high migration, GBM lacks an effective treatment strategy and is associated with poor prognosis. SC66 is a novel AKT inhibitor that has been reported to exert antiproliferative activity in many types of cancer cells. However, it remains unclear whether SC66 has antitumor effects in GBM. In this study, we found SC66 obviously suppressed U87 and U251 cell proliferation and EMT- mediated cell migration and invasion. Moreover, SC66 induced GBM cells apoptosis and arrested cell cycle in G0/G1 phase. Furthermore, SC66 also downregulated AKT signaling pathway in a concentration dependent manner. We also found the level of β-catenin nuclear translocation was prominently downregulated after SC66 treatment. Meanwhile, TCF/LEF luciferase report assay indicated that the activity of TCF/LEF was remarkably suppressed. Elevating β-catenin activity by using IM12 rescued SC66 inhibition‐mediated GBM cell proliferation and metastasis. In addition, SC66 showed significantly suppressed the tumorigenicity compared to the control group in the xenograft mouse model. In conclusion, our study demonstrated that SC66 exerts prominently antitumor efficiency in GBM cells *in vivo* and *in vitro* by downregulated AKT/β-catenin pathway.

## Introduction

GBM is the most prevailing, malignant and lethal primary tumor in the adult central nervous system (CNS), accounting for approximately 50% of all gliomas (Cheng et al., 2019). The standard strategy of GBM includes surgical resection, radiotherapy and adjuvant Temozolomide (TMZ) chemotherapy. TMZ was considered to be the most effective drug for treating glioblastoma. Previous studies reported that TMZ treatment can increase median survival from 12.1 months to 14.6 months and 2-year survival from 10% to 26.5% compared with postoperative radiotherapy alone in GBM patients (Grossman et al., 2009; Li et al., 2018). Unfortunately, at least 50% of GBM patients do not respond to TMZ, and prone to acquire resistance after treatment with TMZ, which limit TMZ’s effectiveness (Lee, 2016; Wu et al., 2018). Hence, it’s urgent to develop new drugs and explore new therapeutic strategies to improve the prognosis of GBM patients.

The phosphatidylinositol 3-kinase PI3K/AKT signaling pathway has been reported to be one of the most frequently activated in the majority of human cancers. AKT is considered to be the hub protein of PI3K/AKT signaling pathway, regulating the activity of over 100 downstream substrates (Karar and Maity, 2011; Mundi et al., 2016; Lien et al., 2017). AKT is a serine/threonine protein kinase, plays a crucial role in cell malignant transformation after activating by phosphorylation. AKT activation is considered to be a hallmark of many human cancers, which contributes to cancer development by promoting cell proliferation, suppressing apoptosis, and increasing oncogenic mutation rates (Robey and Hay, 2009; Wu et al., 2019). Successful inhibition of AKT may have a significant antineoplastic effect, therefore, targeting AKT is an important field of oncological therapy.

SC66 is a new allosteric AKT inhibitor and has been reported to promote AKT ubiquitination and inactivation by preventing the binding of pleckstrin homology (PH) domain to PIP3 (Jo et al., 2011). Another study shown that SC66 promoted cervical cancer cell death by disrupting mTOR signaling and glucose uptake (Rader, 2016). SC66 combined with cisplatin showed a more effective suppression of tumor growth in the xenograft model of ovarian cancer mice (Wu et al., 2019). In addition, SC66 suppressed colon cancer cell proliferation and induced apoptosis (Liu et al., 2019). These studies indicated that SC66 might be a potential antitumor drug. However, whether SC66 can exert antitumor activity in GBM cells is indistinct.

In this work, we investigated the antineoplastic activity of SC66 in GBM cells *in vitro* and *in vivo*, and proved that SC66 effectively suppressed the cell proliferation, EMT- mediated cell migration and invasion. It also arrested the cell cycle in G0/G1 phase and induced cell apoptosis. Furthermore, SC66 displayed potential tumor growth reduction in nude mouse model of GBM. In summary, SC66 might be a new drug for suppressing GBM progression.

## Methods

### Drugs and Antibodies

AKT inhibitor SC66 was purchased form MedChemExpress [HY-19832, purity (99.32%), China] and dissolved in dimethyl sulfoxide (DMSO), which was obtained from Servicebio (G5051, Wuhan, China). The GS3K-β inhibitor IM-12was obtained from Selleck (S7566, purity (99.01%), USA). The antibodies included the following: anti-Phospho-AKT [#4060, Cell Signaling Technology(CST), USA], anti-AKT (#4691, CST), anti-Phospho-GSK-3β (#9323,CST), anti-GSK-3β (#12456,CST), anti-Phospho-β-catenin (#9561,CST), anti-cleaved-caspase3 (ab32042, Abcam, UK), anti-β-catenin (ab32572,Abcam), anti-GAPDH (60004-1-Ig,Proteintech,Wuhan, China), anti-Snai1 (13099-1-AP,Proteintech), anti -BAX (50599-2-Ig,Proteintech), anti-Bcl-2 (127891-AP,Proteintech), anti-Cyclin D1(60186-1-Ig,Proteintech), anti-caspase3 (19677-1-AP,Proteintech), anti-MMP2 (10373-2-AP,Proteintech), anti-Vimentin (sc-6260 Santa Cruz Biotechnology, USA). More detailed antibody information was shown in **Additional File 1**.

### Cell Culture

Humans glioblastoma cell lines (U87 and U251) were purchased from the Cell Bank Type Culture Collection of the Chinese Academy of Sciences (Shanghai, China). Cells were all cultured at 37°C under a humidified atmosphere of 5% CO2 by using Dulbecco’s modified Eagle’s medium (DMEM) supplemented with 10% fetal bovine serum (FBS) (Gibco, USA).

### Cell Viability Assay

The ability of SC66 exerts antiproliferative activity was measured with Cell Counting Kit-8 (CCK-8) according to the supplier’s protocol (Dojindo, Beijing, China). U87 and U251 cells were planted into 96-well plate (5,000 cells/well) and treated with 0, 5, 10, 15, 20, 25, 30 umol/L of SC66 for 24 h. Then 10u per well of CCK8 was added and then incubated at 37°C for 1 h. The OD value was measured with microplate reader at 450 nm. Three independent assays were carried out.

### Colony Formation Assay

An approximate number of 500 cells per well were planted into 6-well plate and cultured with 2ml DMEM containing 10%FBS.Then, the cells were treated with 0, 6, 10, and 15 umol/L of SC66 for two to three weeks until there was significant single-cell colony formation. The cells were fixed with 4% paraformaldehyde and stained with 0.5% crystal violet. The counts of colonies were counted using Image J software.

### EdU-DNA Synthesis Assay

Approximately 5x10^3^cells were seeded in 96-well plates containing 100ul DMEM and treated with 0, 6, 10, 15 umol/L SC66 for 24 h to the next day. The cell growth was studied using Cell-Light EdU Apollo567 *In Vitro* Kit (RiboBio, Guangzhou, China). Cells were cultured in medium supplement with 50ul EdU for 2 h, and fixed with 4% paraformaldehyde for 30 min. Subsequently, 100ul of 1X Apollo^®^ reaction cocktail was added and incubated for 30 min, and then counterstained with 1X Hoechst 33342 in dark for 30 min. Fluorescence images of the Hoechst 33342 and EdU were visualized using a fluorescence microscope (Olympus BX51, Japan).

### Wound-Healing Assay

Cells were seeded in 6-well plates and cultured for a certain time to reach 70% confluency. The sterile pipette tip was used to scratch a linear wound. PBS was used to wash away floating cells three times and serum free DMEM was added for further culturing, and then exposed to different concentration of SC66 (0 and 6umol/L) for 24 h. Images were obtained at 0 h, 24 h, and 48 h and captured under a microscope (Olympus BX51, Japan). ImageJ software was used to analyze the wound healing percentage.

### Transwell Assay

Invasion and migration assay were measured by Transwell chamber (Corning, USA). For the invasion assay, the polycarbonate Transwell filters coated with Matrigel (R&D, USA) to form a continuous membrane, a number of 8x10^3^cells treated with 0, 6, 10, or 15 uM of SC66 for 24 h were seeded into the upper chambers. Simultaneously, 200 ul serum-free DMEM was added into the top chambers, and 600 ul DMEM supplement with 10%FBS was added into the lower chamber. Transwell chambers were placed in an incubator (37°C,5% CO_2_) for 24 h and fixed in 4% paraformaldehyde for 30 min. The non-invasive cells in the upper chambers were moved with cotton swabs, and the cells on the lower chamber were stained with 0.5% crystal violet for 15 min. Air dried and the results were counted under an inverted microscope (Olympus BX51, Japan).

### Cell Cycle Assay

Cells were harvested with 0.25 trypsin after treated with 0, 6, 10, or 15 of SC66 for 24 h. Next, cells were fixed in 70% cold ethanol at −20°C for 12 h. Then the fixed cells were washed three times with PBS and incubated with PBS containing RNase for 30 min. Eventually, the cells stained with propidium iodide (PI) under dark conditions for 15 min. Cell cycle results were measured by FACS Calibur flow cytometer (BD Biosciences, USA) and the data were quantified using ModFit LT 5.0 software.

### Apoptosis Assay

Annexin V-PE/7- ADD kit (Becton Dickinson, USA) were used to measure the apoptosis of glioma cells. Cells were seeded in a 6-well plate and treated with 0,6,10 or 15 uM of SC66 for 24 h. According to the manufacturer’s instruction, the fixed cells were suspended in 1ml 1X binding buffer, and stained with Annexin V-PE/7- ADD for 10 min under dark conditions. For each experiment, 2x10^5^ cells were analyzed FACS Calibur flow cytometer (BD Biosciences, USA). Early apoptosis and late apoptosis were summed and the total apoptosis rate was calculated.

### Western Blot Analysis

Cells treated with 0, 6, 10, or 15 uM of SC66 for 24 h, and then lysed in RIPA buffer (Beyotime, China) on ice for about 30 min. The cell lysate was centrifuged at 1.2x10^5^ rpm for 15 min at 4°C and protein concentrations were quantitatively determined by BCA method (Beyotime, China). The lysate was mixed with loading buffer and heated at 100 °C for 10 min. The protein was loaded onto a 10% or 12% SDS-PAGE and transferred to a PVDF membrane (Millipore, Germeny). Next PVDF membrane was blocked in 5% non-fat milk for 1 h, and membranes were immunoblotted with primary antibodies to Phospho-AKT, Phospho-GSK-3β, Phospho-β-catenin, AKT, GSK-3β, β-catenin, GAPDH, BAX, Bcl-2, cleaved- caspase3, casapse3, snai1, MMP2, vimentin and Cyclin D1 with an appropriate dilution concentration overnight at 4°C. Subsequently, the membranes were incubated with secondary antibodies (Antgene, Chian,1:10,000) at dark condition for 1 h. The membranes were visualized with Odyssey (LI-COR biosciences, USA).

### Immunofluorescence Staining

The sterilized slides were placed in a 6-well plate, and approximately 3x10^4^ cells were planted per well. After 12 h of sc66 treatment, they were fixed in 4% paraformaldehyde for half an hour and penetrated with 0.5% Triton X-100 at room temperature for 10 min, and then blocked with 1% Albumin Bovine V (Service bio, China) for 30 min at room temperature. Cells were incubated with primary antibodies overnight at 4°C. The next day, cells were washed and then incubated with secondary antibody (Antgene, China) for 1 h under dark conditions. Subsequently, cells nuclei were counterstained with diamidino-phenyl-indole (DAPI) (Antgene, China) in dark conditions for 5 min. The images were captured under a fully automatic Microscope (Olympus BX63; Japan).

### Immunohistochemistry Staining

The tumor tissues from nude mouse models were fixed in 4% paraformaldehyde and embedded in paraffin. The sections were deparaffinized, hydrated and antigen repaired with 10mM sodium citrate (pH, 6.0). After removing endogenous peroxidase by using 3% H_2_O_2_, the samples were blocked with Albumin Bovine V (Service bio, China) for 30 min. The samples were incubated with primary antibody overnight at 4°C according to the manufacturer’s recommended concentration. The reactions were visualized using a 3,3′-diaminobenzidine visualization kit (Servicebio, China) and counterstained with hematoxylin to visualize nuclei for 1 min. The images were captured under a microscope (Olympus BX51, Japan)

### Luciferase Assays

Cells were seeded in 6-well plates and treated with 0 or 6uM of SC66 for 24 h. The cells were harvested after co-transfection with TCF/LEF1 luciferase reporter plasmid and pGMLR-TK luciferase reporter plasmid (Yeasen Biotech Co, China) for 48 h. The Renilla and firefly luciferase activities was assessed by the Dual Luciferase Reporter Gene Assay Kit (Yeasen Biotech Co, China) according to the supplier’s protocol

### Animal Experiments

All nude mice were purchased from Shulaibao (Wuhan,China) Biotechnology Co., Ltd. All animal experiments were reviewed and approved by the Animal Ethics Committee of Wuhan University People’s Hospital. U87 cells in the logarithmic growth stage were resuspended in PBS at a concentration of 5x106 cells/100μL and then subcutaneously injected into the armpits of 5-week-old Balb/c nude mice. When the tumor volume reached about 100 mm^3^, the mice were randomly divided into two groups (n = 8) and injected intraperitoneally with SC66 (25 mg/kg) every 3 days for 6 consecutive times. Meanwhile, the control group was intraperitoneally injected with the same volume of DMSO. In addition, we measured tumor volume every 3 days. Tumor volume was calculated using the formula V = L ×W^2^ × 1/2 (V, volume; L, length; W, width). Two days after the last drug treatment, the mice were killed and the tumor was stripped. Half of each tumor was fixed with 4% paraformaldehyde and paraffin-embedded sections, the other half was extracted for western blot.

### Statistical Analysis

Statistical analyses were performed using GraphPad Prism 8.0 software. All experimental results were presented as mean ± standard deviation (SD). Student’s t-test was used to analyze the differences between two groups. One -way ANOVA test was used for the comparison among three or more groups, and if analysis of variance was significant, testing of differences between groups *via* Tukey’s multiple comparisons test. A p value of less than 0.05 was considered as statistical significance.

## Results

### SC66 Suppresses the Proliferation of Human GBM Cells

**Figure 1:**
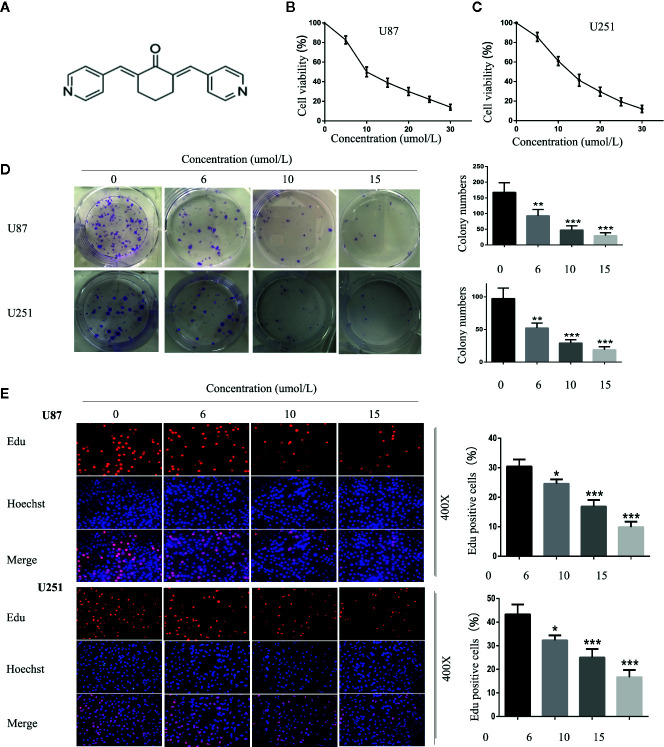
SC66 inhibited the proliferation of GBM cells *in vitro*. **(A)** The molecular structure of SC66. **(B, C)** Cell viability was measured by CCK8 assay after SCC66 treatment with at various concentrations (0, 5, 10, 15, 20, 25, 30 umol/L). **(D)** SC66 inhibited U87 and U251 cells colony formation and results were statistically analyzed. **(E)** EDU assay shown that SC66 inhibited DNA synthesis in U251 and U87 cells. For at least three independent experiments. *P < 0.05, **P < 0.01, ***P < 0.001.

SC66 is an allosteric inhibitor which displays a dual-inhibitory function toward AKT activity. Its chemical formula is shown in **Figure 1A**. To investigate the antiproliferative activity of SC66 against GBM cells, U87 and U251 cells viability were detected using CCK-8. The results showed that SC66 inhibited the proliferation of U87 and U251 cells in a dose-dependent manner. The IC50 values of SC66 in U87 and U251 cells were 10 umol/L and 12 umol/L respectively (**Figures 1B, C**). Besides, the results of colony formation assay also indicated that lesser and smaller colonies were formed as the treatment concentrations of SC66 increased in both GBM cell lines (0, 6, 10, and 15 umol/L) (**Figure 1D**). In addition, results of EdU-DNA synthesis assay showed that SC66 induced a prominent decrease in the percent of EdU-positive cells in U87and U251 cells (**Figure 1E**). These results indicated SC66 inhibited the proliferation of GBM cells in a dose-dependent manner.

### SC66 Inhibits the EMT of GBM Cells

**Figure 2:**
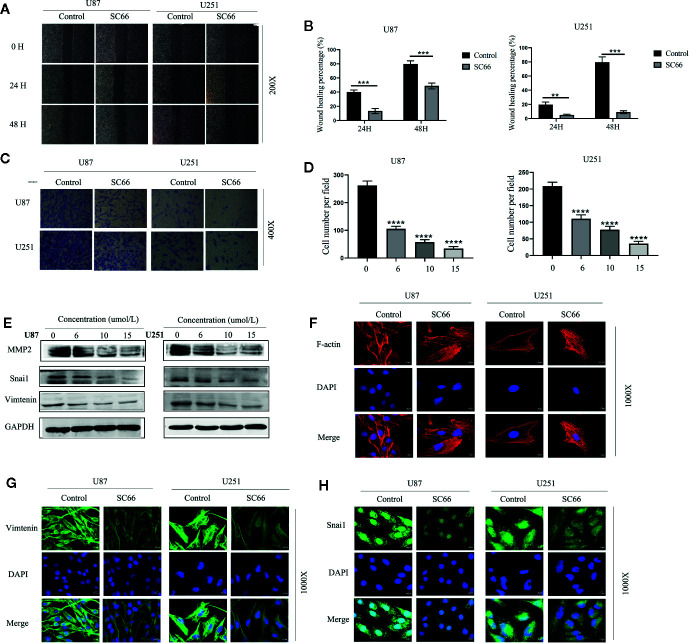
SC66 suppressed the metastatic biological functions of GBM cells *in vitro*. **(A, B)** Wound healing was demonstrated that SC66 suppressed the migration of U87 and U251 cells. **(C, D)** Tanswell assay showed that SC66 inhibited the invasion of U87 and U251 cells. **(E)** The expression of MMP2, Vimentin, and Snai1 after SC66 treatment. **(G, H)** Representative fluorescent images of Vimentin and Snai1 in U87 and U251 cells. **(F)** Cells were stained of F-actin with Phalloidin. For at least 3 independent experiments. **P < 0.01, ***P < 0.001, ****P < 0.0001.

We stained the cytoskeleton with phalloidin, SC66 treatment obviously altered the morphology of U87 and U251 cells, and the cytoskeleton was also damaged (**Figure 2F**). Epithelial-to-mesenchymal transition (EMT) is a biological process that allows epithelial cells to obtain mesenchymal phenotypes. During this process, epithelial cells undergo morphological and biochemical changes to reorganize their cytoskeleton (Colella et al., 2019).

Furthermore, EMT was confirmed to be important for promoting tumor progression and metastasis in various cancers (Han et al., 2017). Therefore, we hypothesized that SC66 might inhibit EMT processes. In order to further verify our conjecture, wound healing and Transwell assays were performed to assess the inhibitory effect of SC66 on migration and invasion of GBM cells. As shown in **Figure 2A**, compared to SC66 -treated group, the untreated group demonstrated a rapid increase in the gap distance. The wound healing percentage remarkably higher in untreated group after 48 h (**Figure 2B**). The result revealed that SC66 impeded the migration of GBM cells in comparison to control SC66-free cells. Similarly, Transwell assays indicated a dose-dependent manner decrease in invasion of SC66-treated GBM cells (**Figures 2C, D**). Meanwhile, western blotting showed that the expression of invasion-related protein Matrix metalloproteinase 2(MMP2), snail1 and vimentin was decreased after SC66 treatment (**Figure 2E**; **Additional File 2: Figures S1A, B**). Simultaneously, immunofluorescence assays demonstrated that the expression of Vimentin and Snai1were reduced in U87 and U251cells after SC66 treatment (**Figures 2G, H**). These changes in Vimentin and Snail indicated that a mesenchymal-to-epithelial transition (MET) occurred in U87 and U251cells after SC66 treatment, which is reverse process of EMT. All of results *in vitro* suggested that SC66 could significantly block EMT of U87 and U25.

### SC66 Arrests Cell Cycle at G0/G1 Phase in GBM Cells

**Figure 3:**
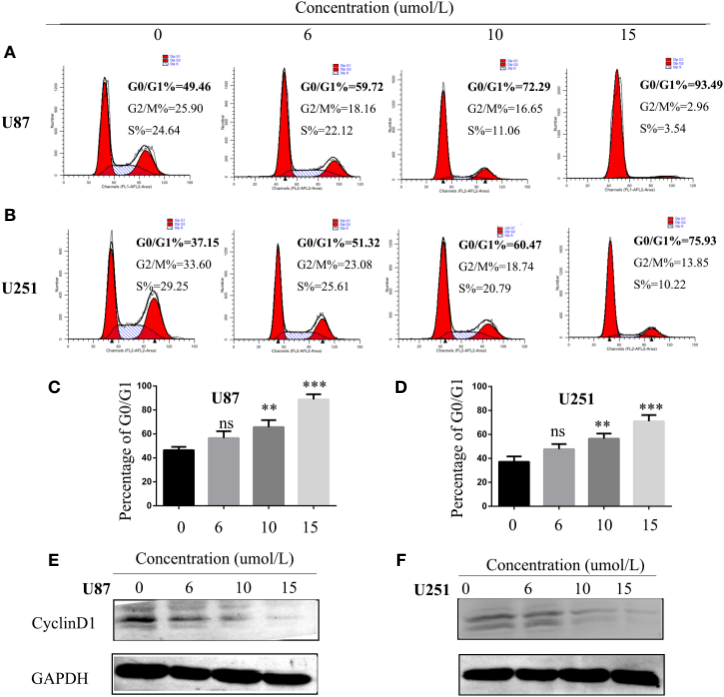
SC66 arrests cell cycle at G0/G1 phase in U87and U251cells. **(A–D)** Cells accumulated at the G0/G1 phase after SC66 treatment for 24 h measured by flow cytometry and results were statistically analyzed. **(E, F)** Cell cycle protein expression quantified with western blot. For at least three independent experiments. n.s., no significance; **P < 0.01, ***P < 0.001.

One of the reasons for the infinite proliferation of tumor cells is the deregulation of the cell cycle. We hypothesized that SC66 inhibits GBM cell proliferation by blocking the cell cycle. In our study, flow cytometry analysis was utilized to verify whether SC66 affects cell cycle distribution. The results revealed that SC66 treatment resulted in accumulation of cell cycle in G0/G1 phase (**Figures 3A–D**). In order to explain underlying mechanism of G0/G1 arrest induced by SC66, we investigated the expression of key cell cycle regulatory proteins by western blot, the expression of Cyclin D1 was decreased with the increase of the treatment dose of SC66 (**Figures 3E, F**; **Additional File 2: Figure S1C**).

### SC66 Induces Cell Apoptosis in U87 and U251 Cells

**Figure 4:**
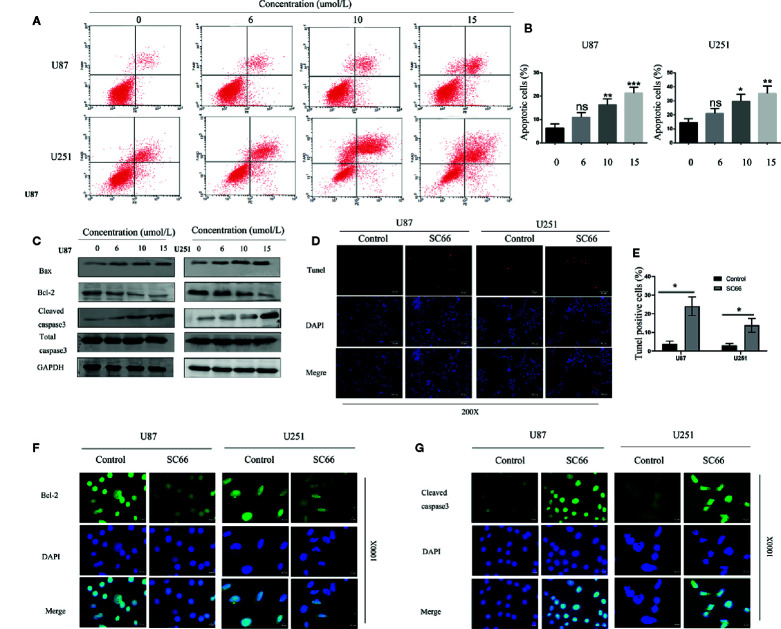
SC66 induced apoptosis of GBM cells *in vitro*. **(A, B)** Cells apoptosis were remarkably enhanced after SC66 treatment for 24 h measured by flow cytometry and results were statistically analyzed. **(C)** Cell apoptosis-related protein expression quantified with western blot. **(D, E)** U251 and U87 cells death were detected by TUNEL staining. **(F, G)** Representative fluorescent images of Bcl-2 and Cleaved- caspase3 in U87 and U251 cells. For at least three independent experiments. n.s., no significance; *P < 0.05, **P < 0.01, ***P < 0.001.

We further investigated the effect of SC66 on cell apoptosis. Flow cytometry analysis results demonstrated that a dose-dependent manner increased in U87 and U251 apoptosis cells after SC66 treatment for 24 h (**Figures 4A, B**). Further western bolt analysis indicated that Bax and cleaved caspase3 expression levels were strikingly reduced, and the expression of Bcl2 was remarkably increased (**Figure 4C**; **Additional File 2: Figures S1D, E**). We evaluated cell apoptosis by performing TUNEL staining assays, and the results showed that TUNEL-positive cells proportion was higher after SC66 treatment (**Figures 4D, E**). In addition, immunofluorescence staining assays also demonstrated that Bcl 2 expression level was reduced and cleaved caspase 3 was upregulated (**Figures 4F, G**). These results suggested that SC66 induced cells apoptosis in U87and U251cells.

### SC66 Inhibits AKT/β-Catenin Signaling Pathways in U87 and U251 Cells

**Figure 5:**
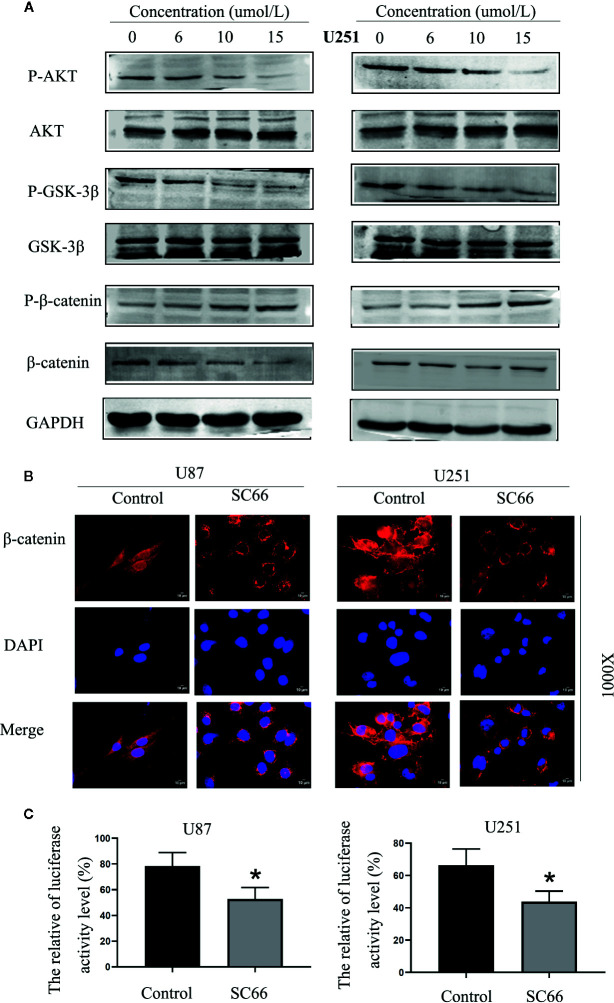
SC66 inhibits AKT/β-catenin signaling pathways in U87 and U251 cells. **(A)** Western blot assay of main protein expression of signaling pathways after SC66 treatment. **(B)** Representative fluorescent images of β-catenin in U87 and U251 cells. Nuclear transfer level of β-catenin was remarkably decreased after SC66 treatment compared with control group. **(C)** The relative TCF/LEF luciferase activity is shown as the percentage of relative light units of firefly luciferase to Renilla luciferase. For at least three independent experiments. *P < 0.05.

SC66 is a novel small molecule allosteric inhibitor. To further confirm whether SC66 exerts anti-tumor effects by affecting the AKT signaling pathway, the related proteins of AKT pathway was verified by western blot, including AKT, GSK3-β, β-catenin and their corresponding phosphorylated forms. The results demonstrated that p-AKT, p-GSK-3βand β-catenin were remarkably reduced with a dose- dependent manner, and p-β-catenin was notably accumulated. However, total AKT and GSK3-β expression were not significant change (**Figure 5A**; **Additional File 2: Figures S1F, I**). Furthermore, immunofluorescence assay revealed that nuclear transfer level of β-catenin was remarkably decreased after SC66 treatment compared with control group (**Figure 5B**). TCF/LEF luciferase reports showed that the TCF/LEF luciferase activity in U87 and U251 cells were significantly reduced after SC66 treatment (**Figure 5C**). The results may demonstrate that SC66 inhibits AKT/β-catenin signaling pathways in U87 and U251 cells.

### Elevating β-Catenin Activity Rescued SC66 Inhibition‐Mediated GBM Cell Proliferation and Metastasis

**Figure 6:**
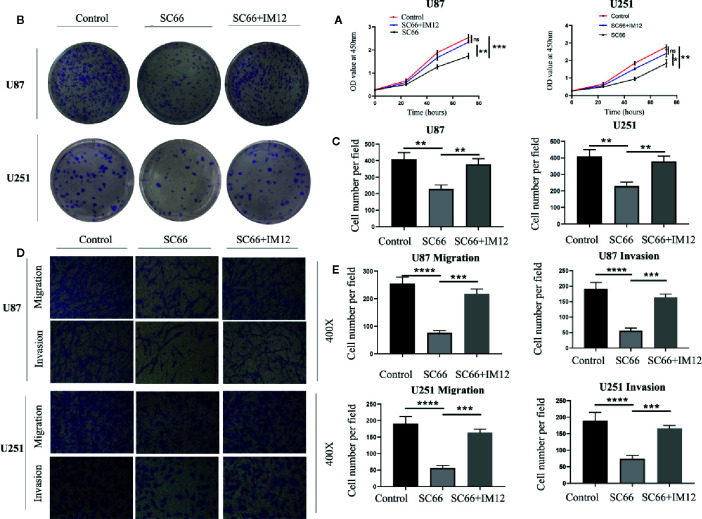
Elevating β-catenin activity rescued SC66 inhibition‐mediated GBM cell proliferation and metastasis. **(A–C)** IM12 was used to enhance β-catenin signaling pathways. CCK 8 and Clone formation assays were performed to investigate the proliferation in U87 and U251 cells. **(D, E)** Transwell assay was performed to detect the migration and invasion in GBM cells. For at least three independent experiments. n.s., no significance; *P < 0.05, **P < 0.01, ***P < 0.001, ****P < 0.0001.

In order to further confirm SC66 exerts antineoplastic effect through the AKT/β-catenin signaling pathways. IM12 which an inhibitor of GSK-3β that has been confirmed to enhances canonical Wan/β-catenin signalling (Schmöle et al., 2010). IM-12 was added to cells after 24 h of SC66 treatment. CCK 8 assays showed that IM12 rescued cell proliferation ability inhibited by SC66, and colony formation assay also confirmed the same results (**Figures 6A–C**). In addition to, Transwell assays indicated the SC66 inhibited function of migration and invasion was partly abrogated after IM12 elevated Wan/β-catenin signaling in U87 and U251cells (**Figures 6D, E**). These results indicated that SC66 mediated β-catenin signaling activation to inhibit GBM malignancy.

### SC66 Suppressed Tumor Growth in Nude Mouse Models

**Figure 7:**
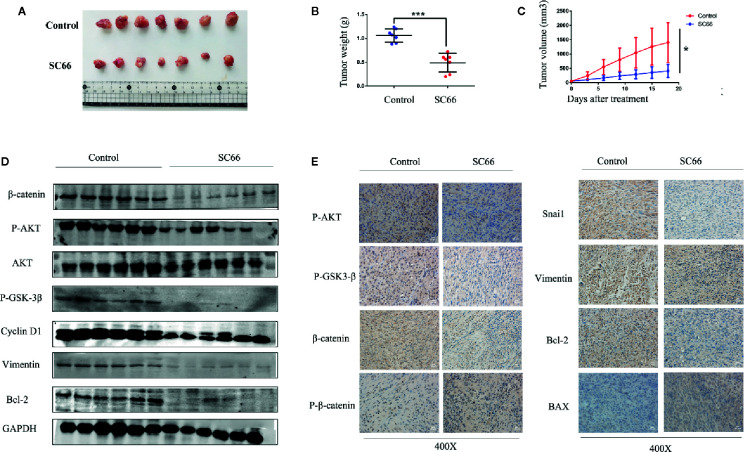
SC66 suppressed tumor growth in nude mouse models. **(A)** Image of the xenograft tumors formed in nude mouse models. **(B, C)** Tumor weight and tumor volume were calculated. **(D)** Western blot assay of main protein expression of control group and SC66-treated group. **(E)** Representative images of IHC staining of P-AKT,P-GSK-3β, β-catenin, P-β-catenin, Snai1, MMP2, Bax, Bcl-2, and Cleaved-caspase3. *P < 0.05, ***P < 0.001.

To assess the anti-tumor ability of SC66 *in vivo*, we established a U87 xenograft tumor model in nude mouse. The results demonstrated that SC66 remarkably suppressed tumor growth, as tumor weights and volume were notably smaller in SC66 treatment group than control group (**Figures 7A–C**). Furthermore, western blot results indicated that β-catenin, P-AKT,P-GSK-3β, Cyclin D1, Vimentin, and Bcl-2 protein expression in xenograft tumor were obviously reduced in SC66 treatment group (**Figure 7D**; **Additional File 2: Figure S1J**). Moreover, immunohistochemical staining revealed that the expression of P-AKT, P-GSK-3β, β-catenin,Snai1,Vimentin and Bcl-2 were significantly decreased, while the expression of BAX, and P-β-catenin was remarkably increased (**Figure 7E**). Immunofluorescence assay also demonstrated that the expression of P-AKT and β-catenin were notably decreased in SC66 treatment group (**Additional File 3: Figure S2**).

## Discussion

Gliomas are the most common malignant brain tumors in adult, and it is estimated that the annual incidence in the United States is 6.6 per 100,000 individuals. About half of newly diagnosed gliomas are classified as glioblastoma (GBM), which is the most aggressive subtype with the worst prognosis among all gliomas (Reifenberger et al., 2017). Therapeutic options are limited, including surgery and treatment with radiotherapy plus TMZ chemotherapy. Due to benefits from TMZ chemotherapy, survival of GBM patients prolong for ~2.5 months (Wang et al., 2016). Nevertheless, the median survival time of GBM patients is less than 16 months even with optimal treatments (Joseph et al., 2015)., more effective drugs were needed to improve prognosis.

Previous studies have shown that AKT activation can stimulated cell proliferation and affected cell cycle regulation through multiple downstream targets. AKT dependent phosphorylation of target proteins such as GSK3 may promote cell proliferation by regulating the stability and synthesis of proteins involved in cell-cycle entry. GSK3-mediated phosphorylation of cyclin D and cyclin E, which play a significant role in the transformation of G1 to -S phase cell-cycle transition (Manning and Cantley, 2007). In this study, we demonstrated that SC66 notably inhibited GBM cells proliferation in a dose-dependent manner. Meanwhile, SC66 suppressed tumor growth also was confirmed in nude mouse models. Nevertheless, flow cytometry analysis demonstrated that treatment with SC66 induced remarkably cell cycle arrest at the G0/G1 phase in U87 and U251 cells. The results of western blot proved that cell cycle related protein cyclin D1 was downregulated after SC66 treatment. Based on these results, SC66 may arrest the cell cycle in G0/G1phase to exert antitumor effects.

The EMT is a bioprocess that cells lose their epithelial characteristics and obtain characteristics of mesenchymal cell phenotypes. In this process, the property of cell migration and invasion is significantly upregulated. The EMT converts innocent tumors into invasive, metastatic tumors and exerts a vital role in regulating tumor progression and metastasis (Liu et al., 2017; Sun et al., 2018). In this work, we indicated that the expression of EMT related protein snail and vimentin were decreased after SC66 treatment. Treatment with SC66 obviously damaged the architecture of cytoskeleton. Moreover, we confirmed the GBM cell’s migration and invasion capacities were notably downregulated after SC66 treatment. These data indicate that SC66 suppressed EMT-mediated migration and invasion in GBM cells.

Cell apoptosis is the basic mechanism that maintains the homeostasis between cell proliferation and cell death, and also considered as a defense mechanism against tumorigenesis (Valdes-Rives et al., 2017; Zhang et al., 2018). Apoptosis was mediated by the death-receptor-induced extrinsic pathway and the mitochondria-mediated intrinsic pathway, and both pathways ultimately activate caspase-3 to execute apoptosis. Besides, the members of the anti-apoptotic and pro-apoptotic families, such as Bcl-2 and BAX were exerted significantly effect to mediate the activation of caspases in the mitochondria-mediated pathway (Valdes-Rives et al., 2017). Hence, it is a major direction that may enhance GBM cell apoptosis for development of effective therapeutic drugs. Previous study had demonstrated that SC66 could significantly induced apoptosis by accompanied by inactivating AKT. The expression of Bcl-XL was dramatically decreased when colon cancer cells were treated with SC66 (Liu et al., 2019). Consistent with these findings, we found that SC66 treatment induced U87 and U251 cells apoptosis in a dose-dependent manner detected by flow cytometry.

SC66 treatment of GBM cells induced the expression of cleaved-caspase3 and increased BAX expression. Concurrently, Bcl-2 protein expression was decreased, which revealed that apoptotic effects of SC66 might be associated with the mitochondrial pathway.

We further investigated the effect of SC66 on pathway through which SC66 regulated proliferation, EMT and apoptosis of glioma cells. SC66 inhibited colon cancer cell proliferation through AKT/GSK-3β/Bax axis *in vivo* and *in vitro* (Liu et al., 2019). Meanwhile, SC66 induced changes in cytoskeletal organization and ROS production, resulting in phosphorylated AKT level was notably decreased, and demonstrated that SC66 remarkably inhibit tumor growth *via* AKT/mTOR/β-catenin pathway in hepatocellular carcinoma (Cusimano et al., 2015).

GSK-3β was inactivated by phosphorylation at Ser9 site after Wnt signal pathway activation, leading to β-catenin in the cytoplasm was accumulated. Cytoplasmic β-catenin subsequently transferred into nuclear, and bond with TCF/LEF, which resulted in transcriptional expression of target genes, such as CyclinD1, c-Myc, etc. (Ding et al., 2019). After treatment with SC66, the expression of P-GSK-3β and β-catenin were decreased, and P-β-catenin expression was increased. IF assay demonstrated that the level of β-catenin nuclear translocation was prominently downregulated in SC66 treated group. Meanwhile, TCF/LEF luciferase report assay indicated that the activity of TCF/LEF was remarkably suppressed. Moreover, elevating β-catenin activity by IM12 rescued SC66 inhibition‐mediated GBM cell proliferation and metastasis. These results indicated that SC66 could suppressed the development of GBM cells by downregulated AKT/β pathway.

In conclusion, we verified that SC66 exerts prominently antitumor efficiency in GBM cells *in vivo* and *in vitro* by deregulated AKT/β-catenin pathway. We also demonstrated that SC66 inhibited EMT-mediated cell migration and invasion. Our findings may contribute to its potential applications for the valid treatment of GBM.

## Data Availability Statement

The original contributions presented in the study are included in the article/**Supplementary Material**; further inquiries can be directed to the corresponding author.

## Ethics Statement

The animal study was reviewed and approved by The Institutional Animal Care and Use Committee at Renmin Hospital of Wuhan University.

## Author Contributions

LG and JL performed cell biology experiments, western blot, flow cytometry analysis, immunofluorescence, immunohistochemistry, manuscript preparation and analyzed the statistical data. PX, GD, and BL participated in flow cytometry analysis. FY, YT, QS, and YX participated in western blot. HZ, YQ, HJ, KY, RG, and MH participated in immunofluorescence. QC participated in manuscript preparation. All authors contributed to the article and approved the submitted version.

## Funding

This research was supported by the National Natural Science Foundation of China (NO.81572489 and 81372683). This manuscript has been released as a pre-print at Research Square, (Gao et al., 2020).

## Conflict of Interest

The authors declare that the research was conducted in the absence of any commercial or financial relationships that could be construed as a potential conflict of interest.

## Abbreviations

CNS, central nervous system; EMT, Epithelial-to-mesenchymal transition; GBM, Glioblastoma; TMZ, Temozolomide.
