# Supplementary material for: AKT Inhibitor SC66 Inhibits Proliferation and Induces Apoptosis in Human Glioblastoma Through Down-Regulating AKT/β-Catenin Pathway
Source: Front Pharmacol. 2020 Jul 31;11:1102. doi: 10.3389/fphar.2020.01102 (PMC7411127; doi:10.3389/fphar.2020.01102)
Supplement: Supplementary file 1 [file Table_1.docx]

**Table 1** Details of the antibodies

| Antibodies | Company | Catalog number | Host | Dilutions |
| --- | --- | --- | --- | --- |
| Phospho-AKT | Cell Signaling Technology | #4060 | Rabbit | 1:1000 |
| AKT | Cell Signaling Technology | #4691 | Rabbit | 1:1000 |
| Phospho-GSK-3β | Cell Signaling Technology | #9323 | Rabbit | 1:1000 |
| GSK-3β | Cell Signaling Technology | #12456 | Rabbit | 1:1000 |
| Phospho-β-catenin | Cell Signaling Technology | #9561 | Rabbit | 1:1000 |
| Cleaved-caspase3 | Abcam | ab32042 | Rabbit | 1:1000 |
| β-catenin | Abcam | ab32572 | Rabbit | 1:1000 |
| GAPDH | Proteintech | 60004-1-Ig | Mouse | 1:20000 |
| Snai1 | Proteintech | 13099-1-AP | Rabbit | 1:1000 |
| BAX | Proteintech | 50599-2-Ig | Rabbit | 1:2000 |
| Bcl-2 | Proteintech | 127891-AP | Rabbit | 1:1000 |
| Cyclin D1 | Proteintech | 60186-1-Ig | Rabbit | 1:1000 |
| Caspase3 | Proteintech | 19677-1-AP | Rabbit | 1:1000 |
| MMP2 | Proteintech | 10373-2-AP | Rabbit | 1:1000 |
| Vimentin | Santa Cruz Biotechnology | sc-6260 | Rabbit | 1:1000 |
