# Supplementary figures and images for: AKT Inhibitor SC66 Inhibits Proliferation and Induces Apoptosis in Human Glioblastoma Through Down-Regulating AKT/β-Catenin Pathway
Source: Front Pharmacol. 2020 Jul 31;11:1102. doi: 10.3389/fphar.2020.01102 (PMC7411127; doi:10.3389/fphar.2020.01102)

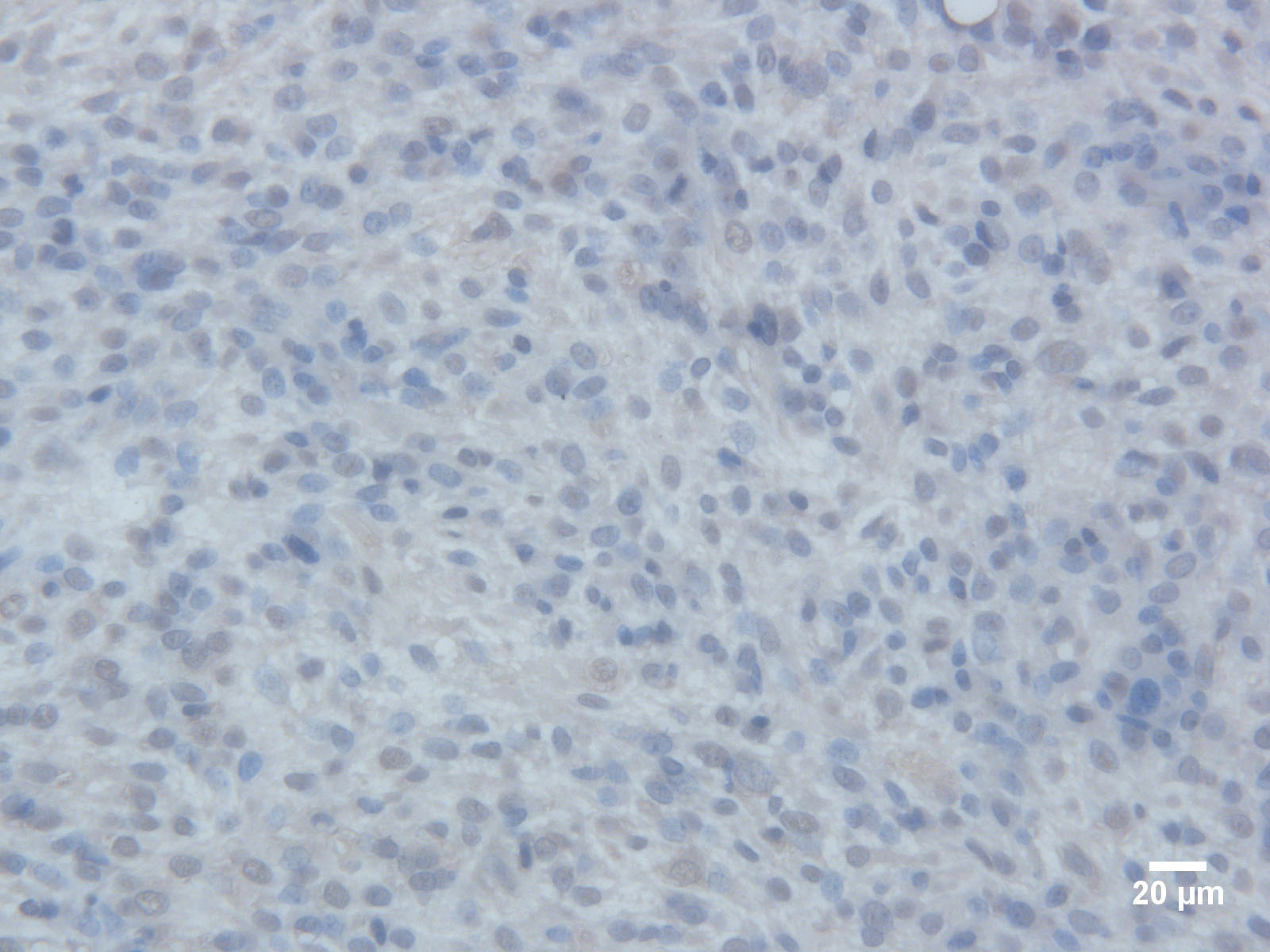

Supplement: Supplementary file 2 [file DataSheet_1.zip › BAX/Control BAX.jpg]

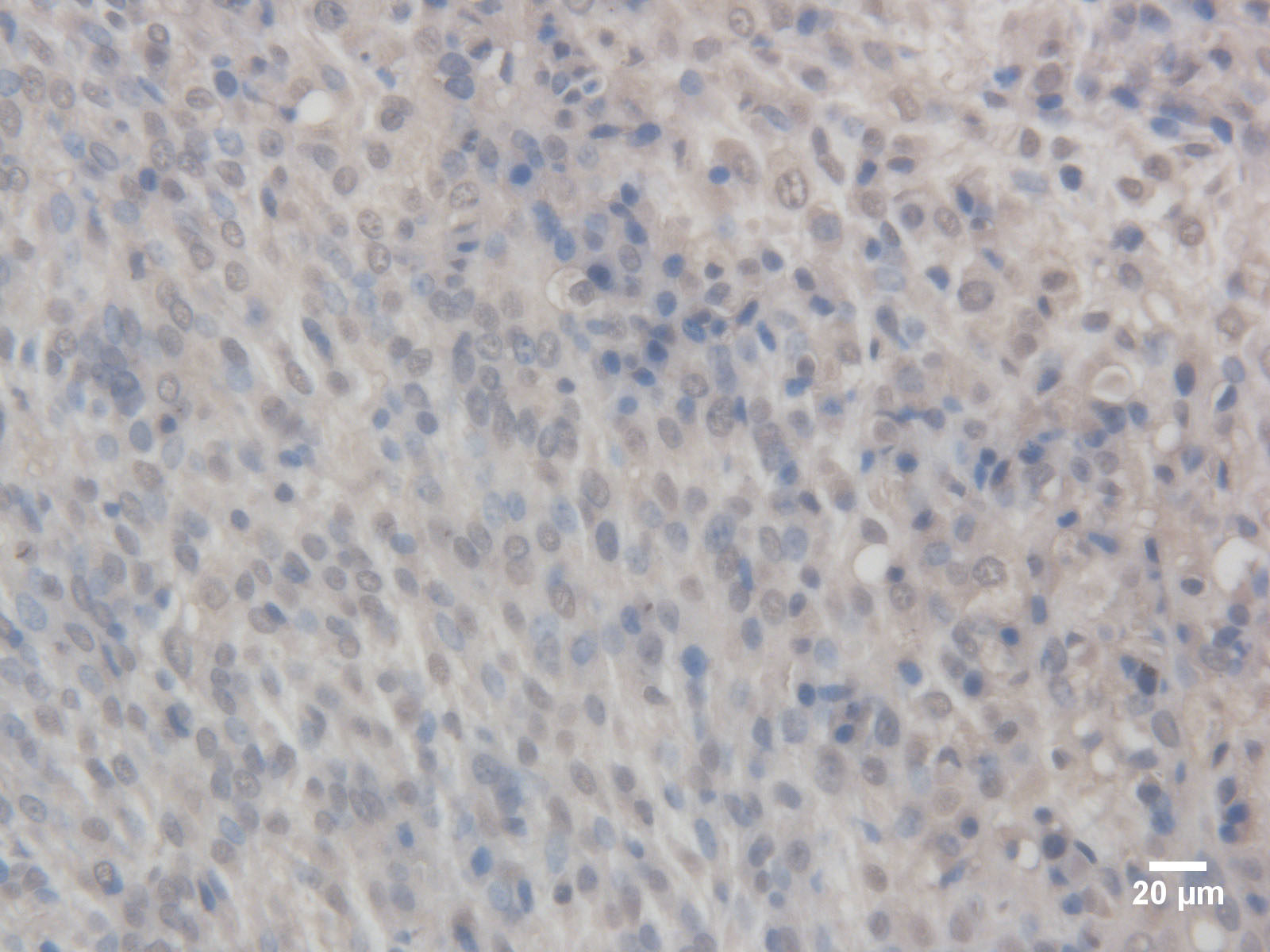

Supplement: Supplementary file 2 [file DataSheet_1.zip › BAX/SC66-BAX.jpg]

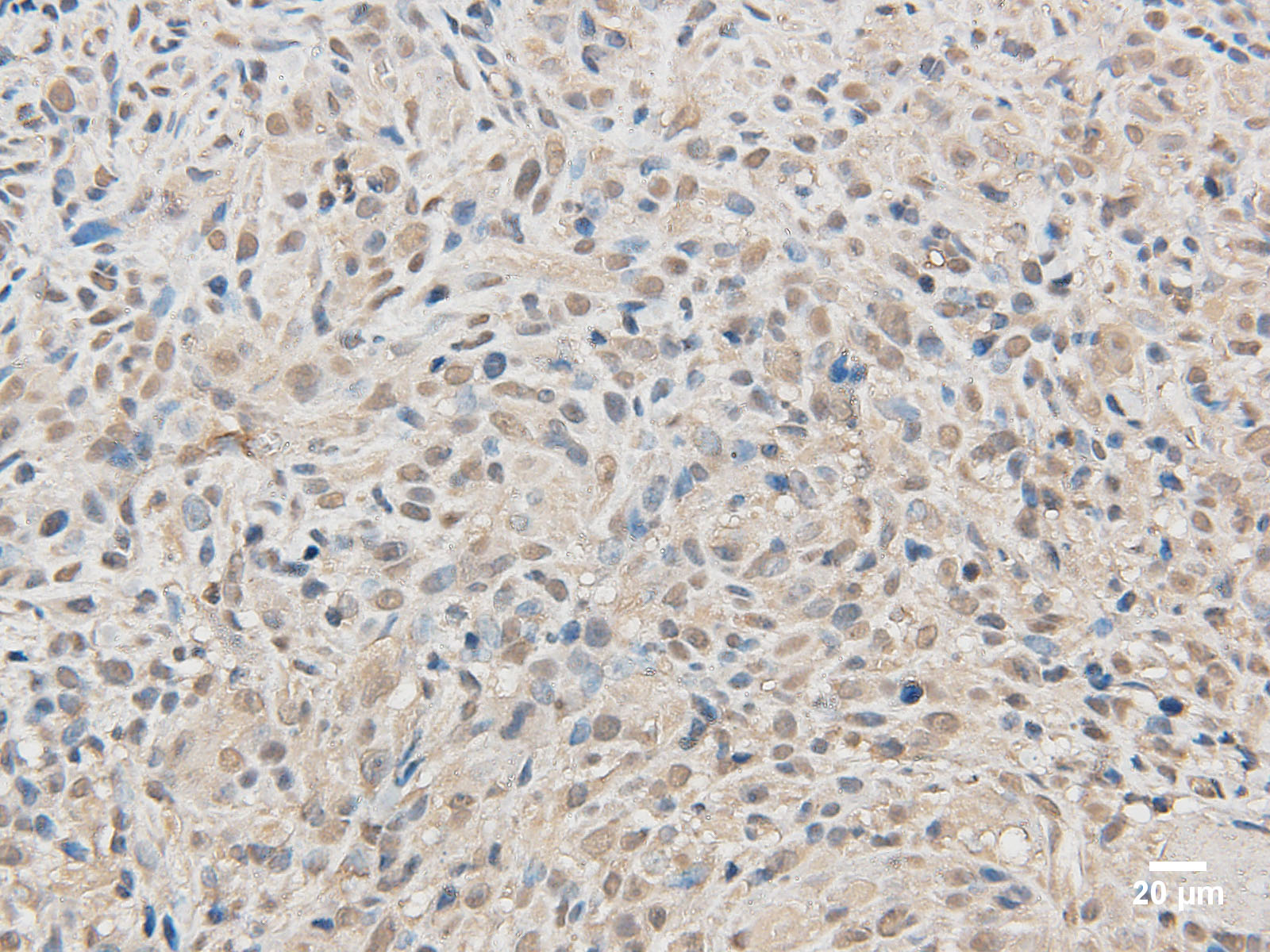

Supplement: Supplementary file 2 [file DataSheet_1.zip › bcl2/Control BAX.jpg]

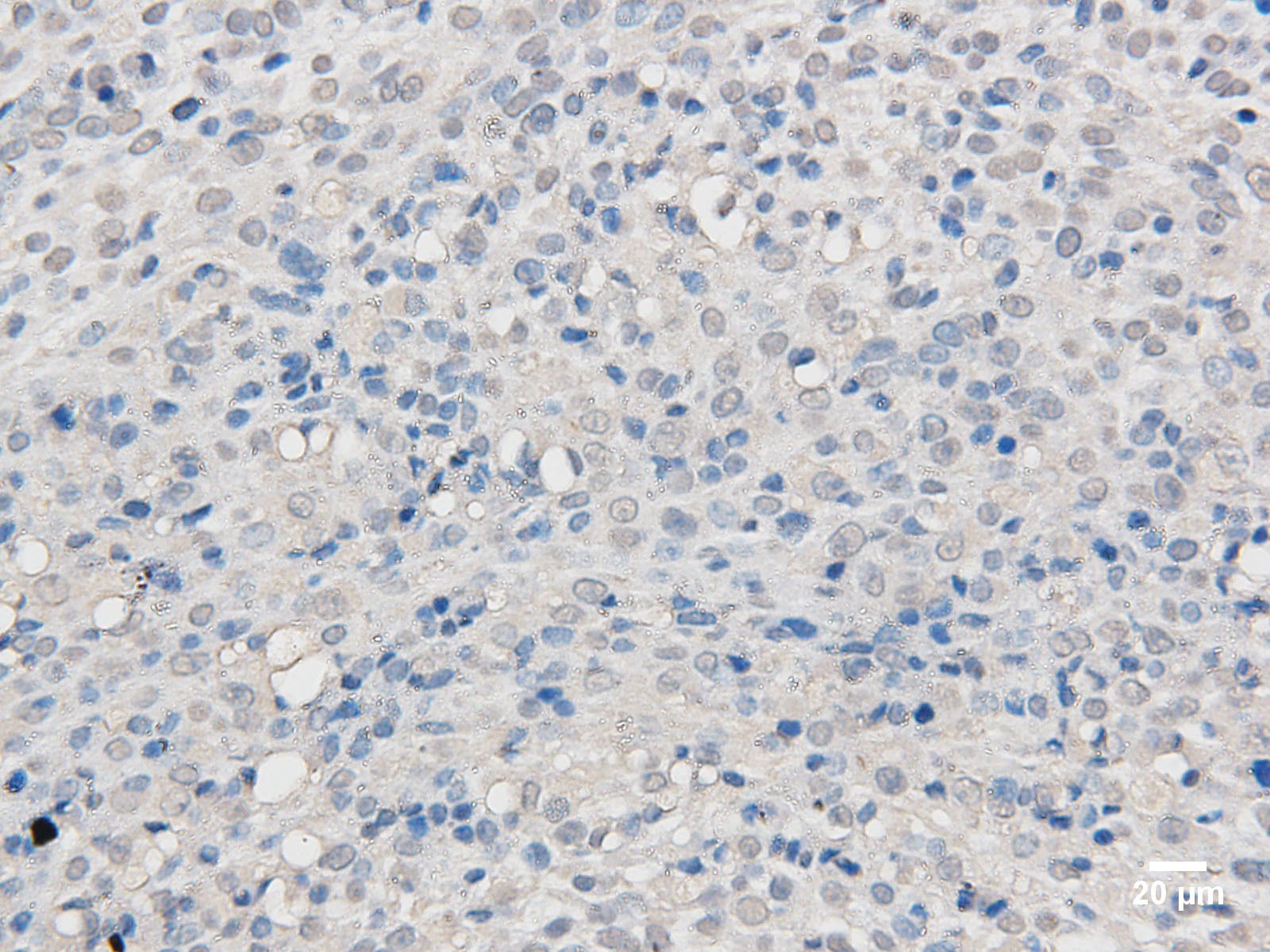

Supplement: Supplementary file 2 [file DataSheet_1.zip › bcl2/SC66 BAX.jpg]

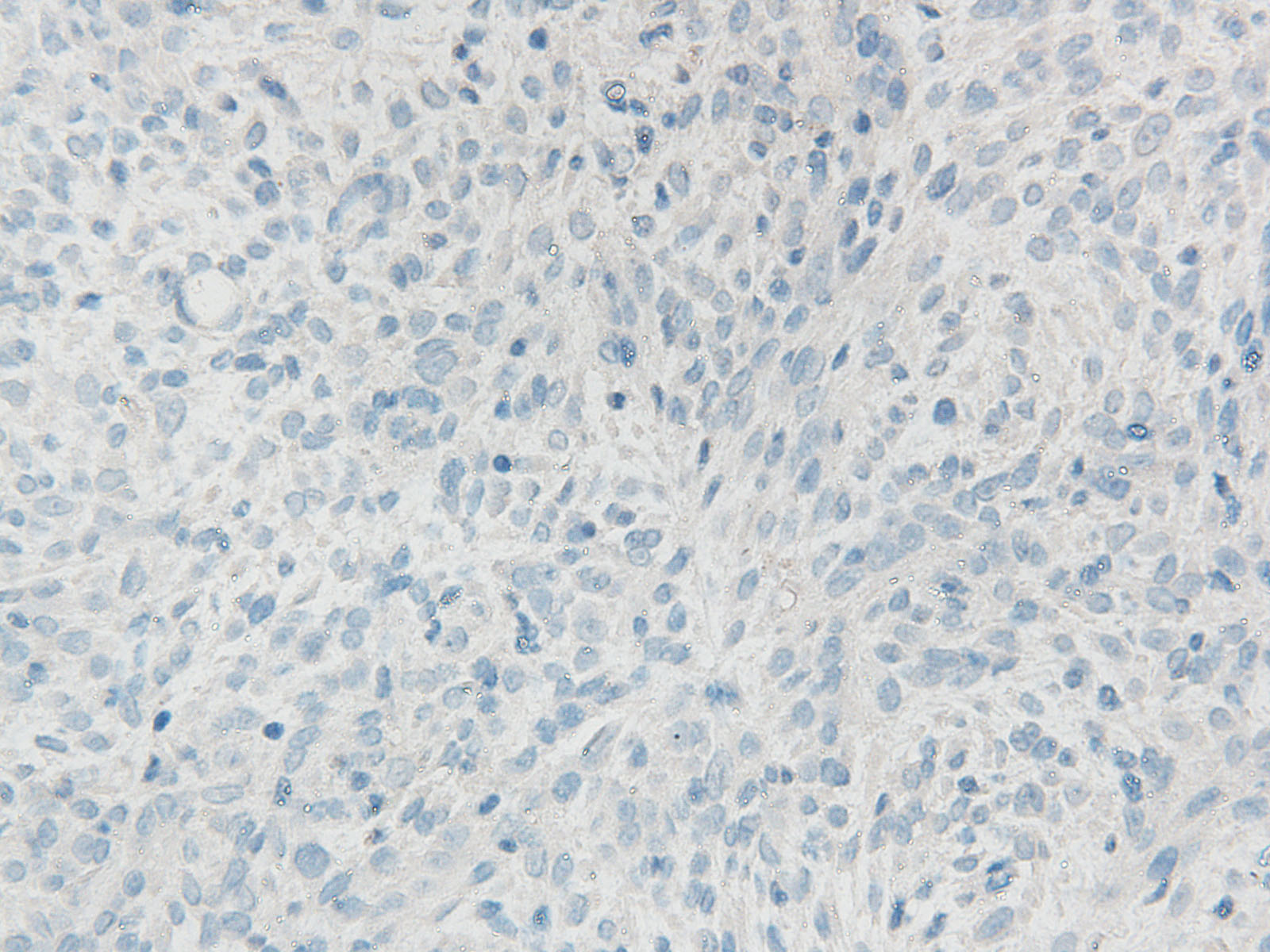

Supplement: Supplementary file 2 [file DataSheet_1.zip › Negative control.jpg]

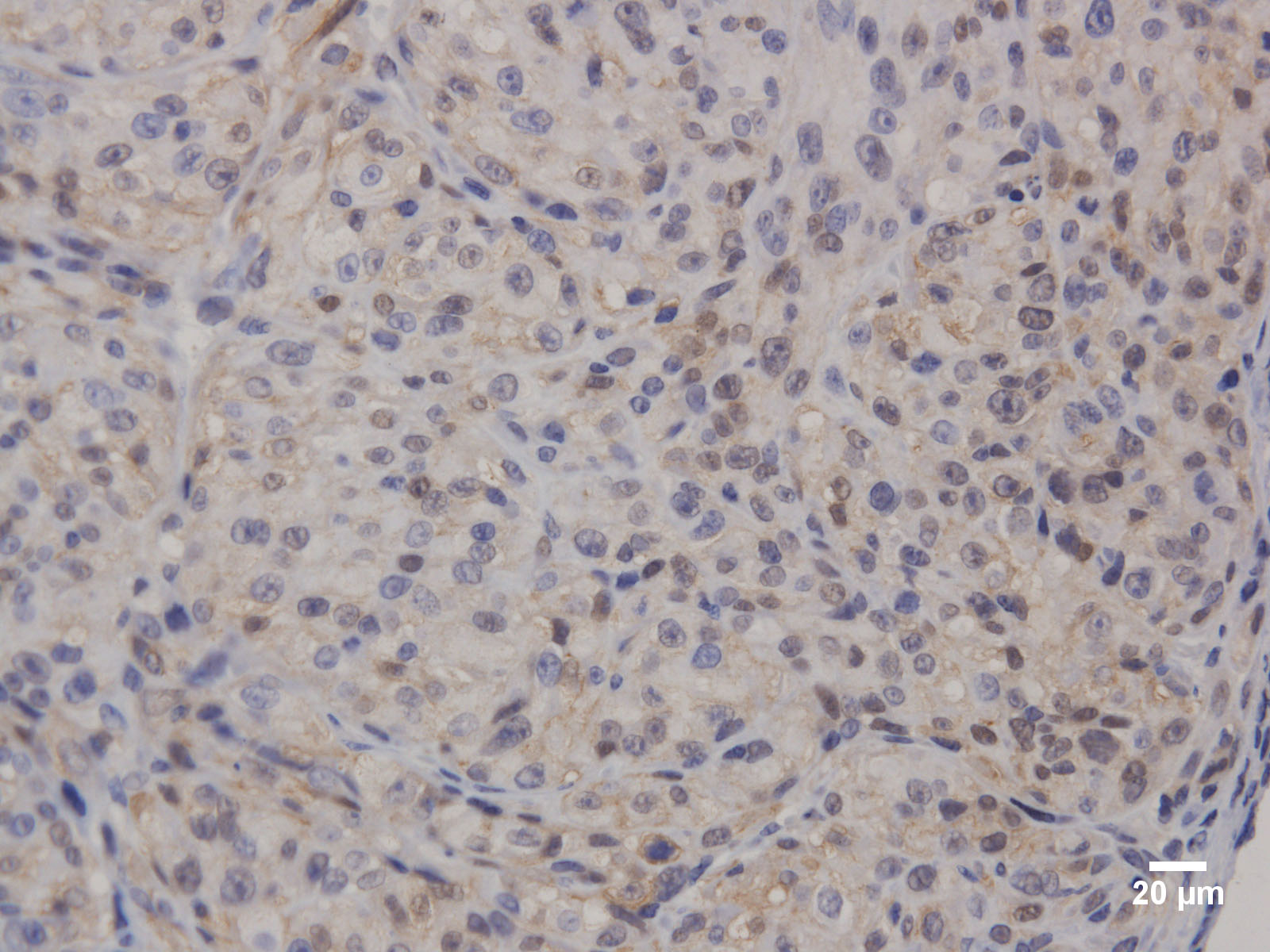

Supplement: Supplementary file 2 [file DataSheet_1.zip › P-AKT/Control P-AKT.jpg]

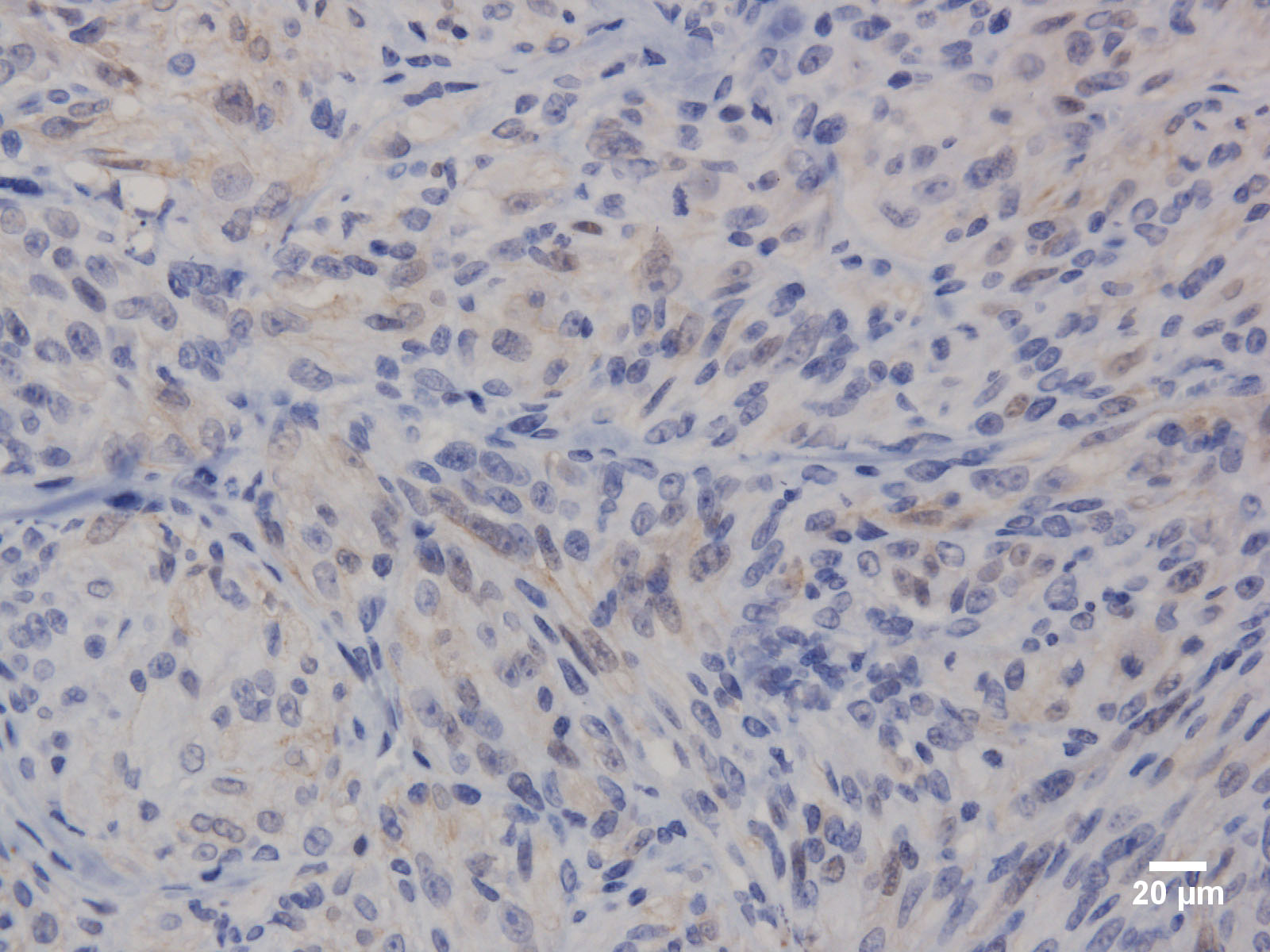

Supplement: Supplementary file 2 [file DataSheet_1.zip › P-AKT/SC66-P-AKT.jpg]

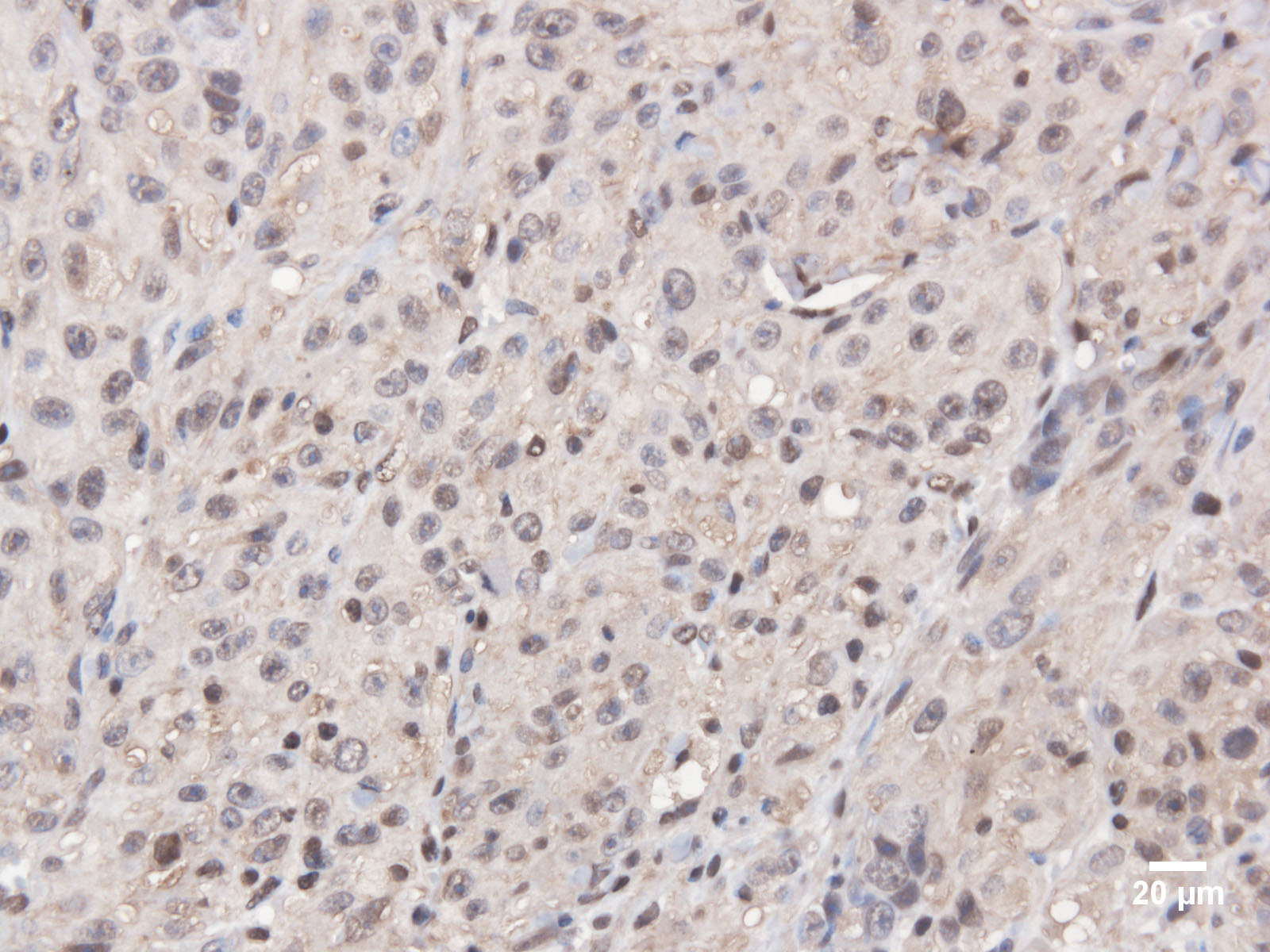

Supplement: Supplementary file 2 [file DataSheet_1.zip › P-GSK3/Control P-GSK-3β.jpg]

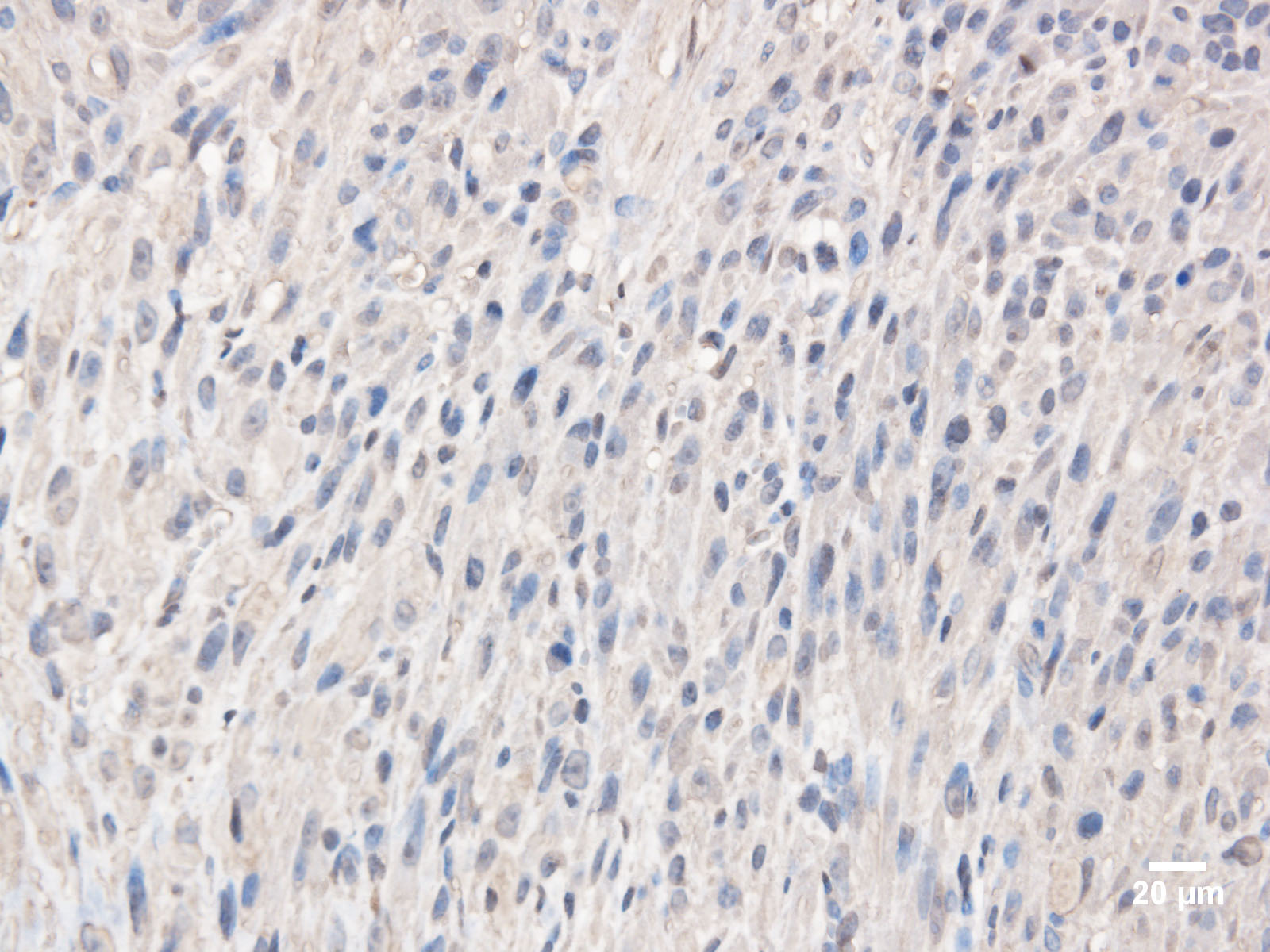

Supplement: Supplementary file 2 [file DataSheet_1.zip › P-GSK3/SC66-P-GSK-3β.jpg]

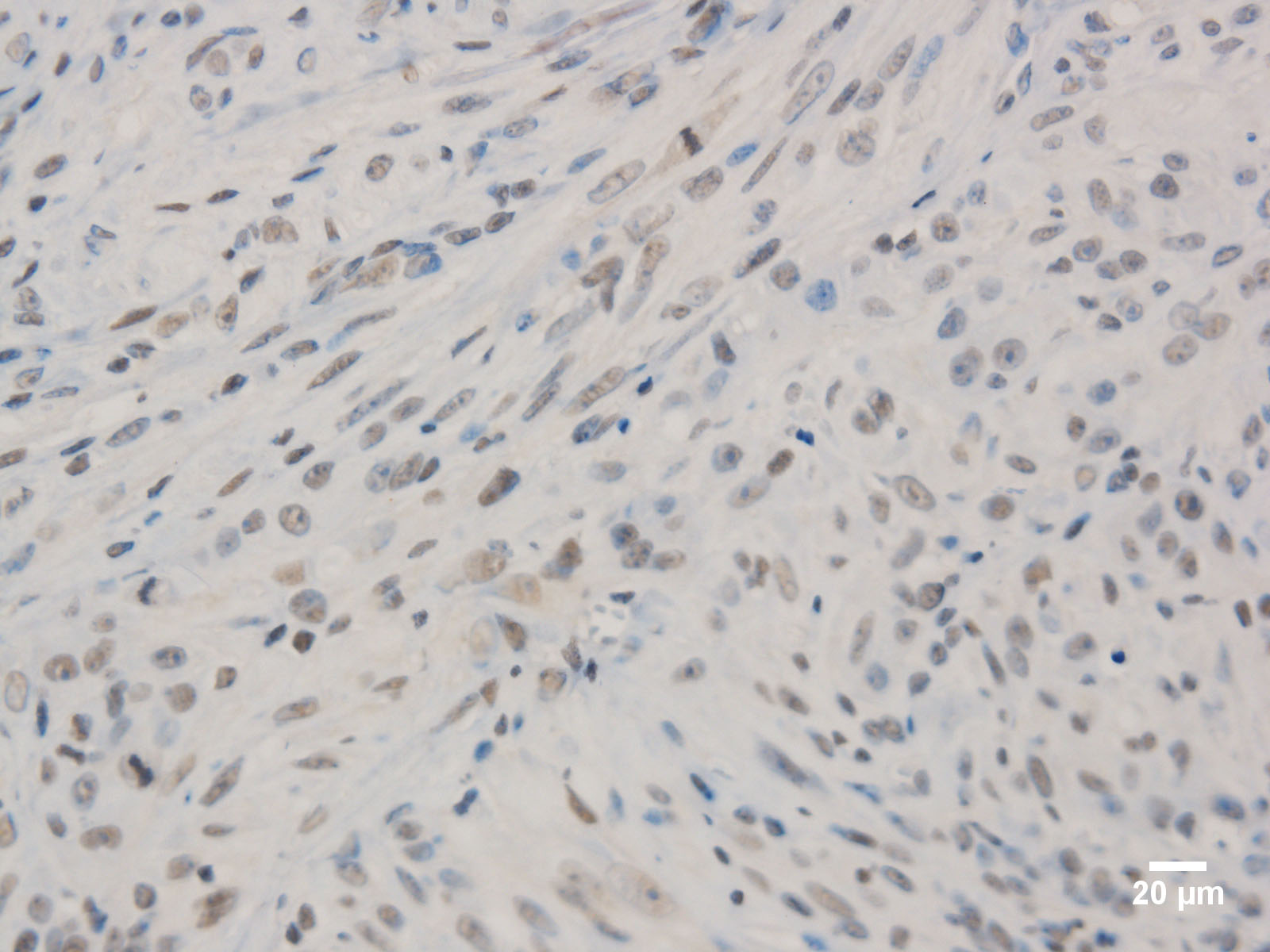

Supplement: Supplementary file 2 [file DataSheet_1.zip › P-β-catenin/Control P-β-catenin.jpg]

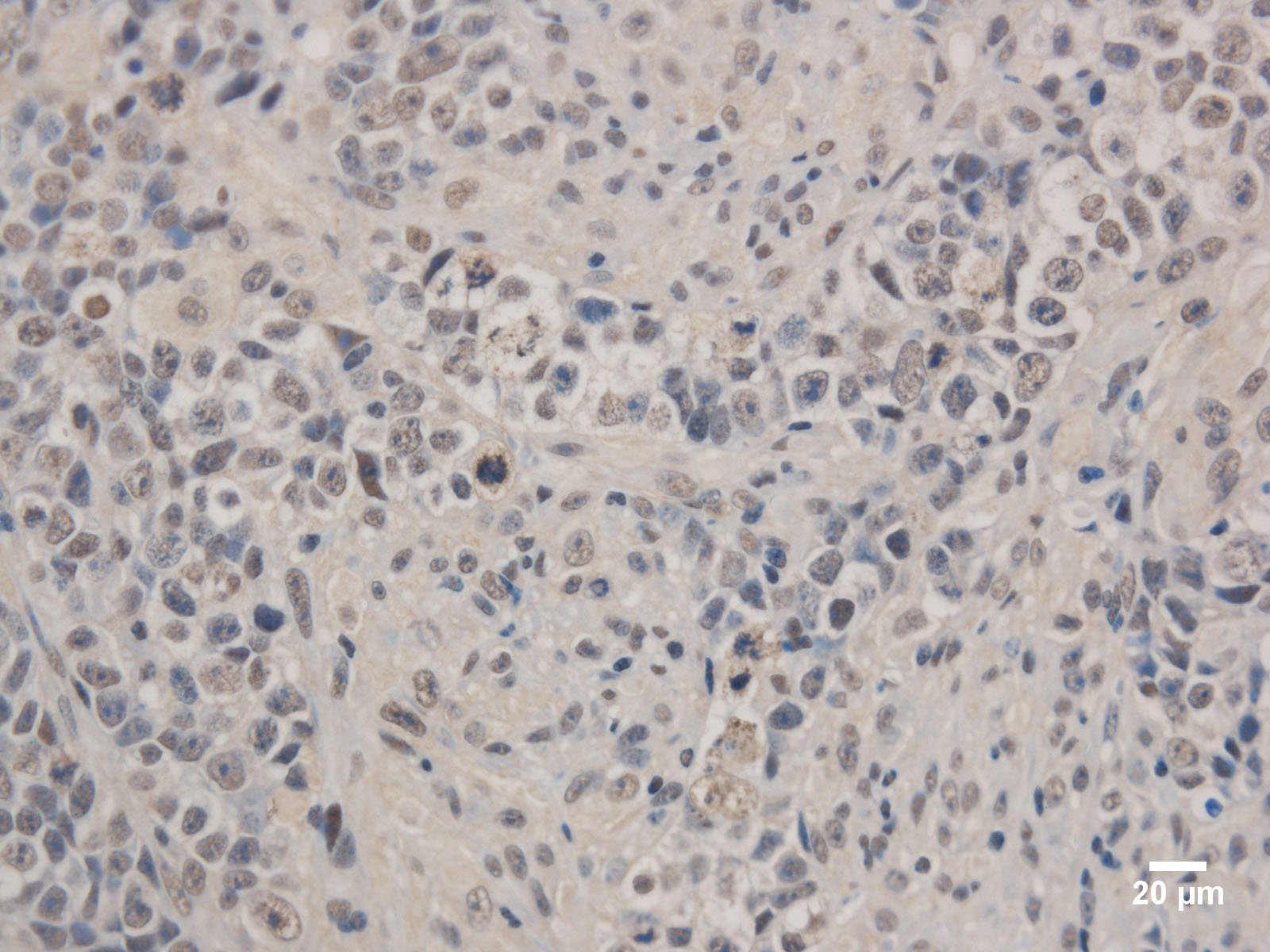

Supplement: Supplementary file 2 [file DataSheet_1.zip › P-β-catenin/SC66-P-β-catenin.jpg]

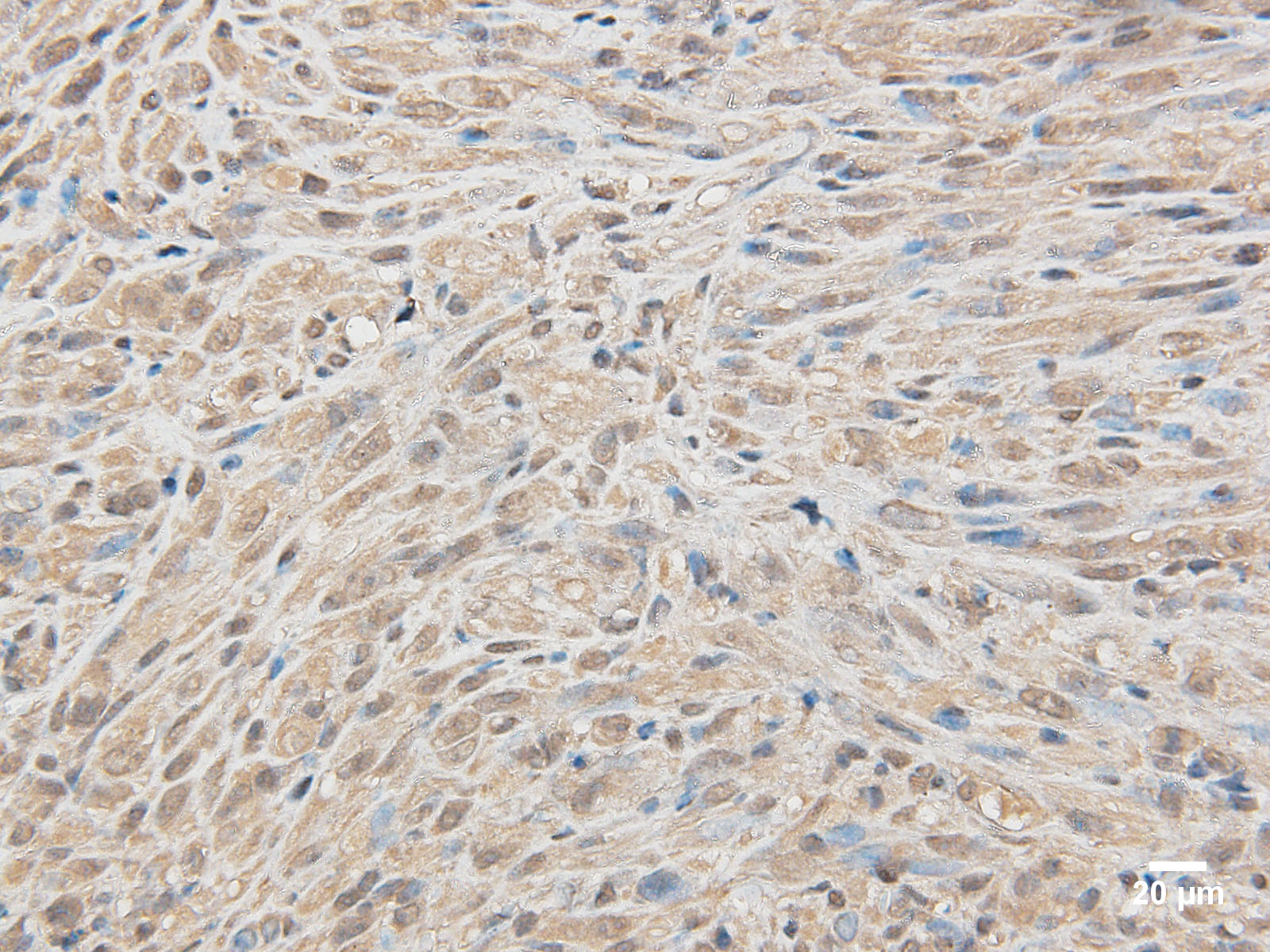

Supplement: Supplementary file 2 [file DataSheet_1.zip › snail/Control Snai1.jpg]

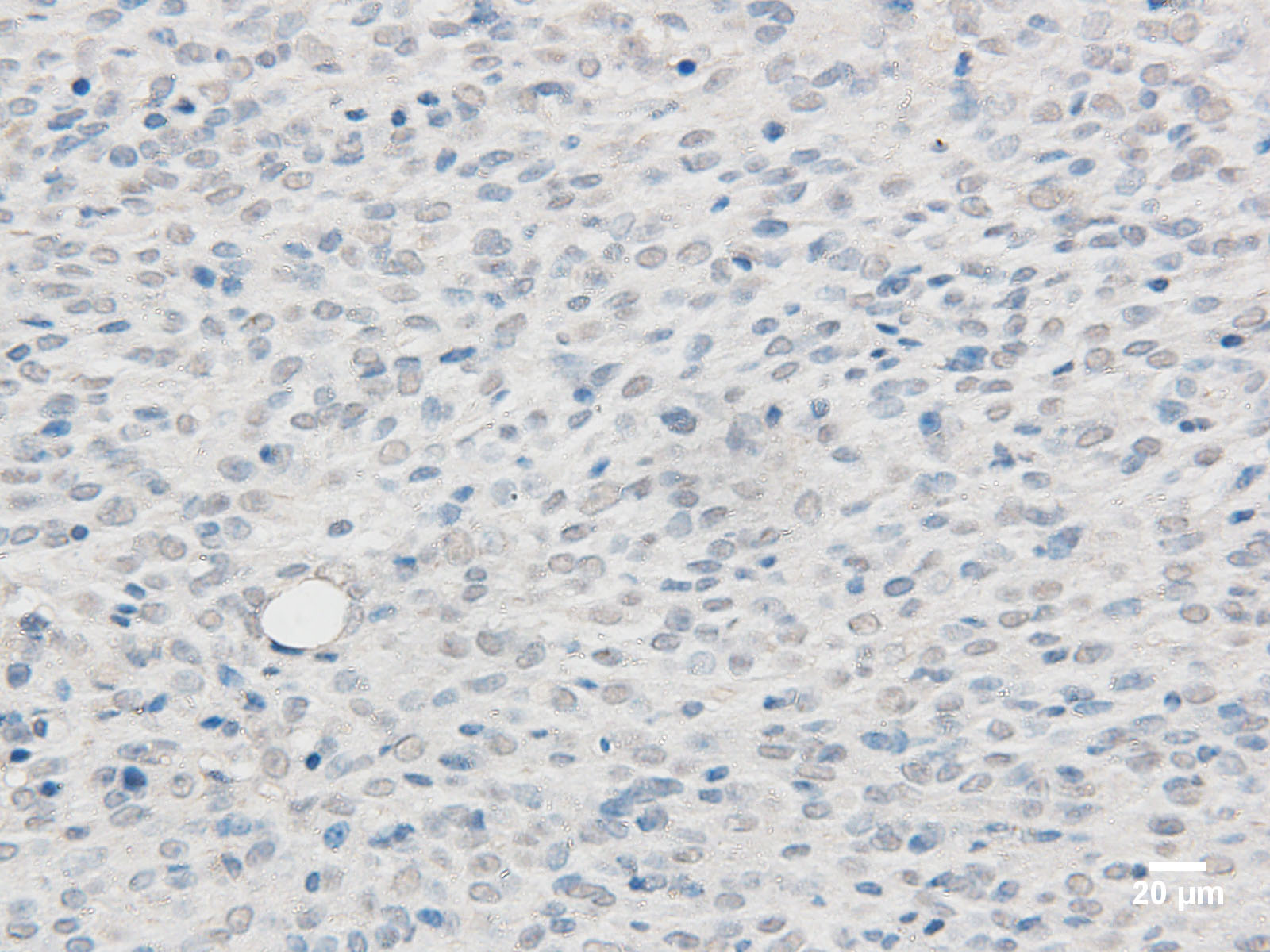

Supplement: Supplementary file 2 [file DataSheet_1.zip › snail/SC66 Snail.jpg]

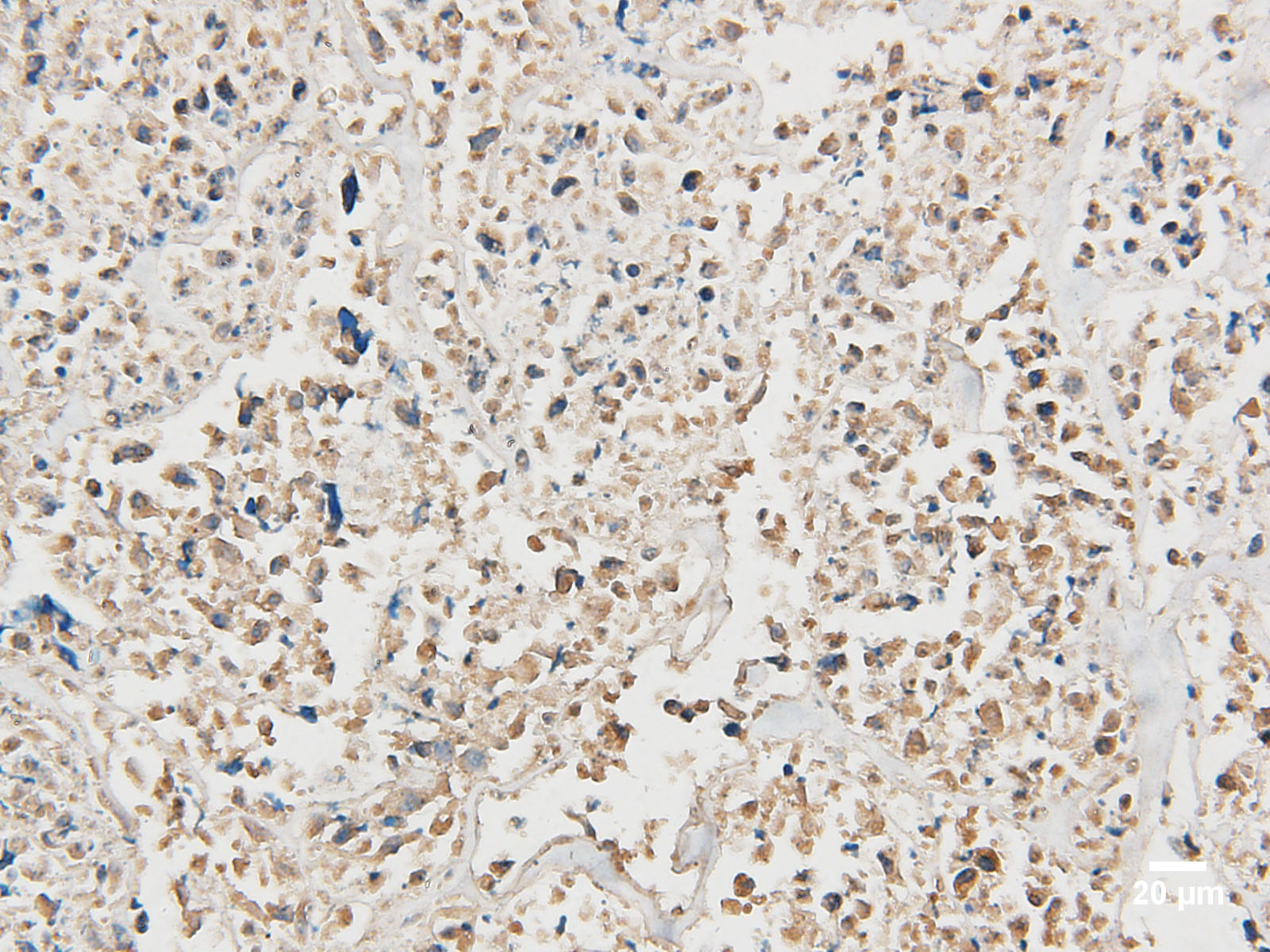

Supplement: Supplementary file 2 [file DataSheet_1.zip › VIM/Control Vimentin.jpg]

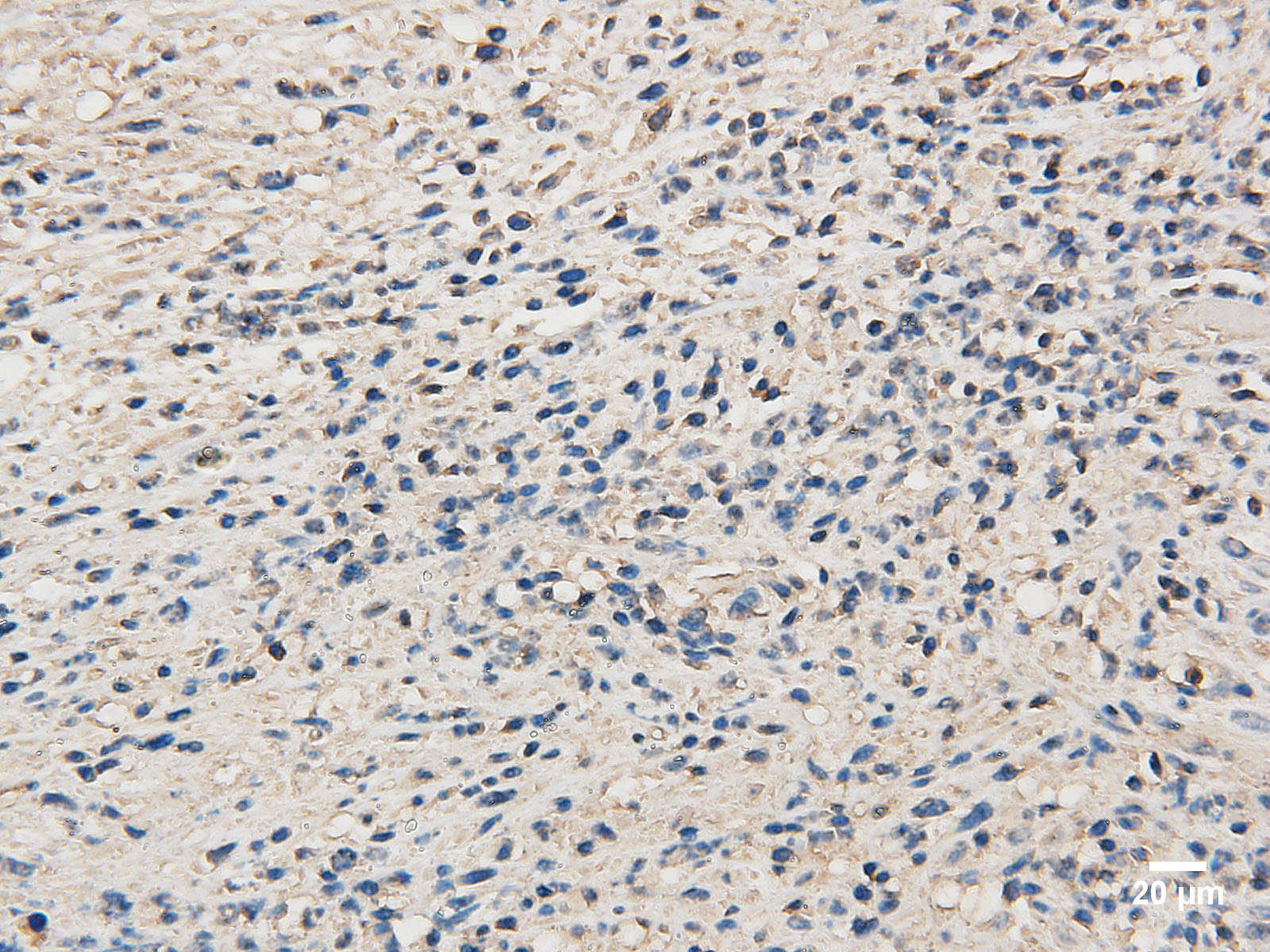

Supplement: Supplementary file 2 [file DataSheet_1.zip › VIM/SC66 Vimentin.jpg]

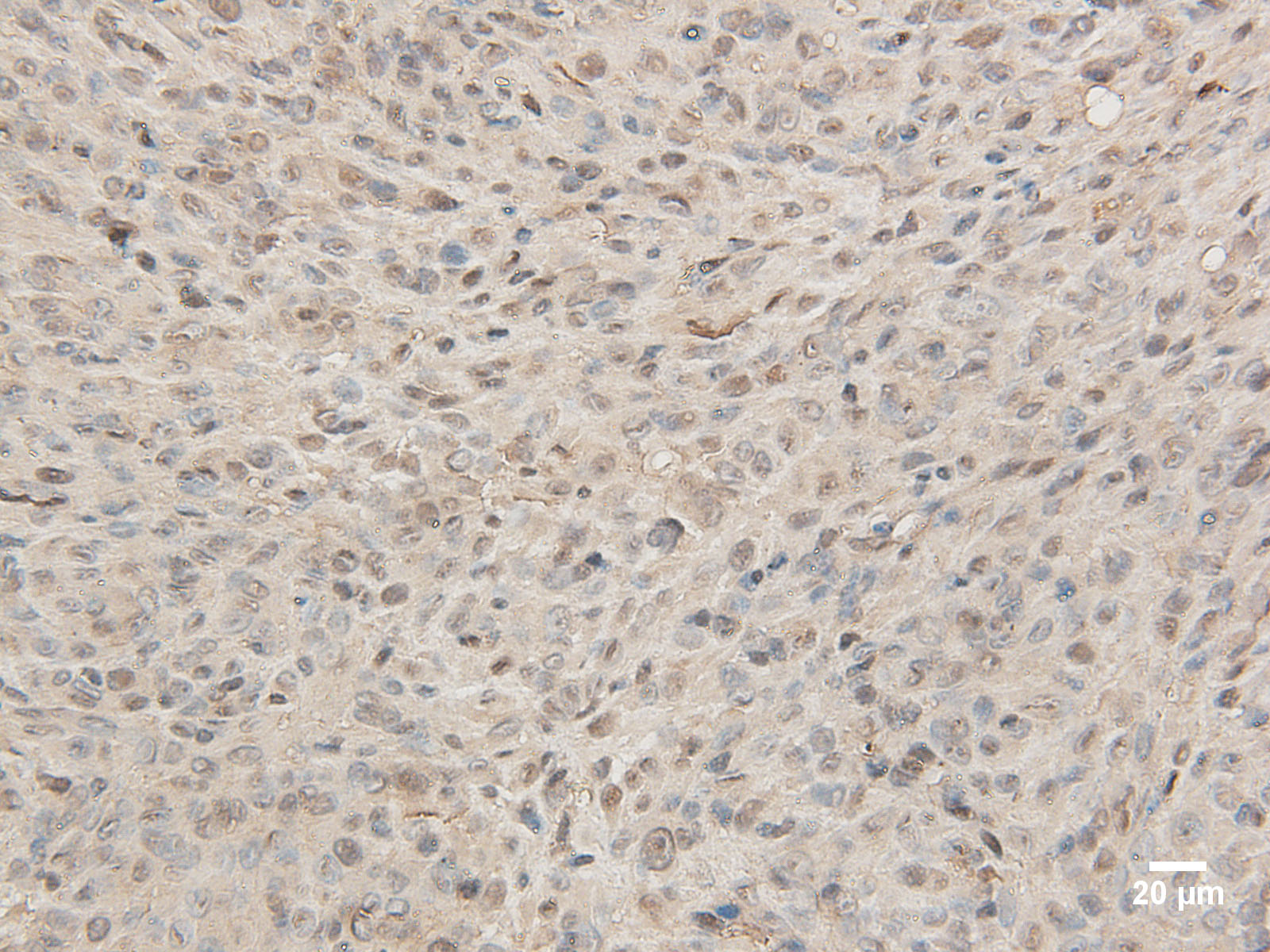

Supplement: Supplementary file 2 [file DataSheet_1.zip › β-catenin/Control -β-catenin.jpg]

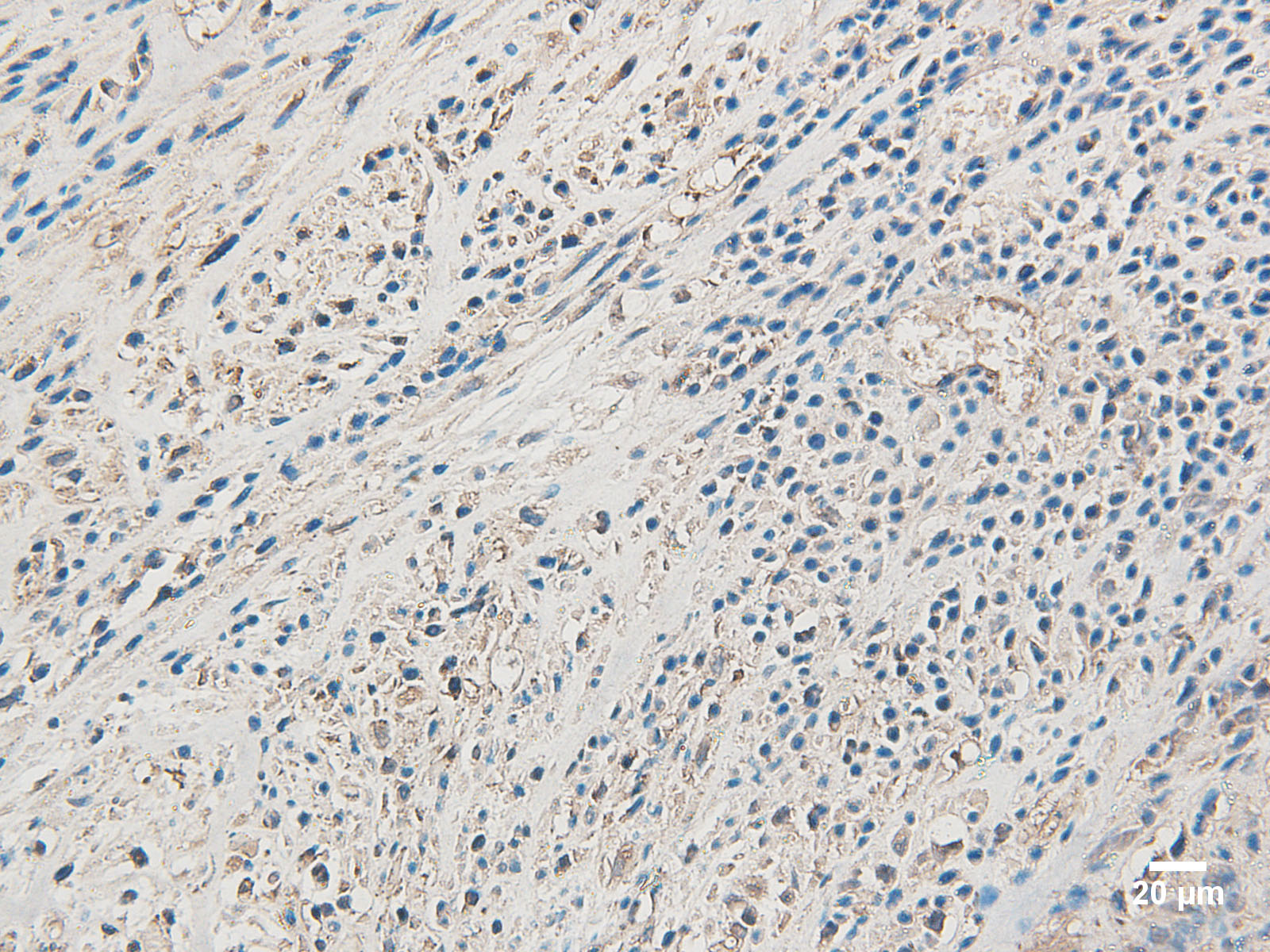

Supplement: Supplementary file 2 [file DataSheet_1.zip › β-catenin/SC66 -β-catenin.jpg]

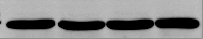

Supplement: Supplementary file 3 [file DataSheet_2.zip › Figure 2/U251/GAPDH-SC66.tif]

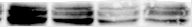

Supplement: Supplementary file 3 [file DataSheet_2.zip › Figure 2/U251/MMP2.tif]

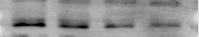

Supplement: Supplementary file 3 [file DataSheet_2.zip › Figure 2/U251/snail.tif]

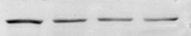

Supplement: Supplementary file 3 [file DataSheet_2.zip › Figure 2/U251/VIMTENIIN.tif]

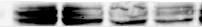

Supplement: Supplementary file 3 [file DataSheet_2.zip › Figure 2/U87/MMP2.tif]

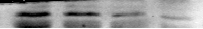

Supplement: Supplementary file 3 [file DataSheet_2.zip › Figure 2/U87/snail.tif]

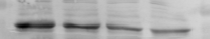

Supplement: Supplementary file 3 [file DataSheet_2.zip › Figure 2/U87/vimtenin.tif]

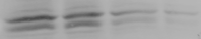

Supplement: Supplementary file 3 [file DataSheet_2.zip › Figure 3/U251/D1.tif]

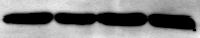

Supplement: Supplementary file 3 [file DataSheet_2.zip › Figure 3/U251/GAPDH.tif]

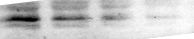

Supplement: Supplementary file 3 [file DataSheet_2.zip › Figure 3/U87/cyclin D1.tif]

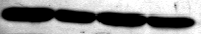

Supplement: Supplementary file 3 [file DataSheet_2.zip › Figure 3/U87/GAPDH.tif]

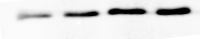

Supplement: Supplementary file 3 [file DataSheet_2.zip › Figure 4/U251/BAX-2tif.tif]

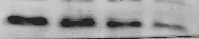

Supplement: Supplementary file 3 [file DataSheet_2.zip › Figure 4/U251/bcl-2-251.tif]

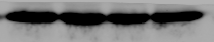

Supplement: Supplementary file 3 [file DataSheet_2.zip › Figure 4/U251/casapse3-2.tif]

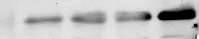

Supplement: Supplementary file 3 [file DataSheet_2.zip › Figure 4/U251/cleaved caspase3-2.tif]

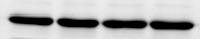

Supplement: Supplementary file 3 [file DataSheet_2.zip › Figure 4/U251/GAPDH-sc66-1tif.tif]

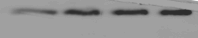

Supplement: Supplementary file 3 [file DataSheet_2.zip › Figure 4/U87/BAX.tif]

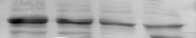

Supplement: Supplementary file 3 [file DataSheet_2.zip › Figure 4/U87/Bcl-2.tif]

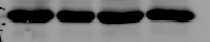

Supplement: Supplementary file 3 [file DataSheet_2.zip › Figure 4/U87/caspase3.tif]

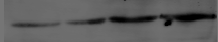

Supplement: Supplementary file 3 [file DataSheet_2.zip › Figure 4/U87/Cleaved caspase3-U87.tif]

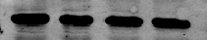

Supplement: Supplementary file 3 [file DataSheet_2.zip › Figure 4/U87/GAPDH-.tif]

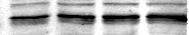

Supplement: Supplementary file 3 [file DataSheet_2.zip › Figure 5/U251/AKT-U87.tif]

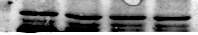

Supplement: Supplementary file 3 [file DataSheet_2.zip › Figure 5/U251/GSK-3β.tif]

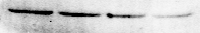

Supplement: Supplementary file 3 [file DataSheet_2.zip › Figure 5/U251/P-AKT.tif]

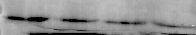

Supplement: Supplementary file 3 [file DataSheet_2.zip › Figure 5/U251/P-GSK-3β.tif]

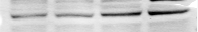

Supplement: Supplementary file 3 [file DataSheet_2.zip › Figure 5/U251/P-β-catenin.tif]

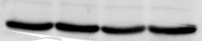

Supplement: Supplementary file 3 [file DataSheet_2.zip › Figure 5/U251/SC66-GAPDH.tif]

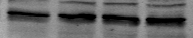

Supplement: Supplementary file 3 [file DataSheet_2.zip › Figure 5/U87/AKT-87.tif]

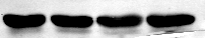

Supplement: Supplementary file 3 [file DataSheet_2.zip › Figure 5/U87/GAPDH.tif]

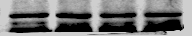

Supplement: Supplementary file 3 [file DataSheet_2.zip › Figure 5/U87/GSK-3β.tif]

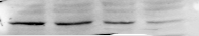

Supplement: Supplementary file 3 [file DataSheet_2.zip › Figure 5/U87/P-AKT.tif]

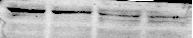

Supplement: Supplementary file 3 [file DataSheet_2.zip › Figure 5/U87/P-GSK-3.tif]

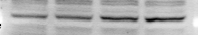

Supplement: Supplementary file 3 [file DataSheet_2.zip › Figure 5/U87/P-β-CATENIN-SC66-1.tif]

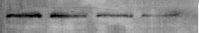

Supplement: Supplementary file 3 [file DataSheet_2.zip › Figure 5/U87/β catenin.tif]

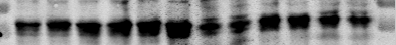

Supplement: Supplementary file 3 [file DataSheet_2.zip › Figure 7/AKT.tif]

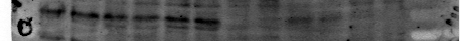

Supplement: Supplementary file 3 [file DataSheet_2.zip › Figure 7/Bcl 2.tif]

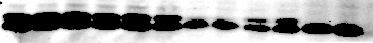

Supplement: Supplementary file 3 [file DataSheet_2.zip › Figure 7/cyclinD1.tif]

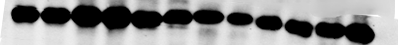

Supplement: Supplementary file 3 [file DataSheet_2.zip › Figure 7/GAPDH-2.tif]

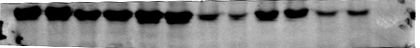

Supplement: Supplementary file 3 [file DataSheet_2.zip › Figure 7/P-AKT.tif]

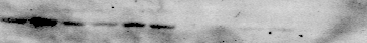

Supplement: Supplementary file 3 [file DataSheet_2.zip › Figure 7/P-GSK-3β.tif]

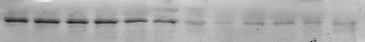

Supplement: Supplementary file 3 [file DataSheet_2.zip › Figure 7/Vim.tif]

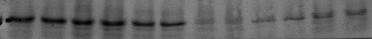

Supplement: Supplementary file 3 [file DataSheet_2.zip › Figure 7/β-catenin-.tif]

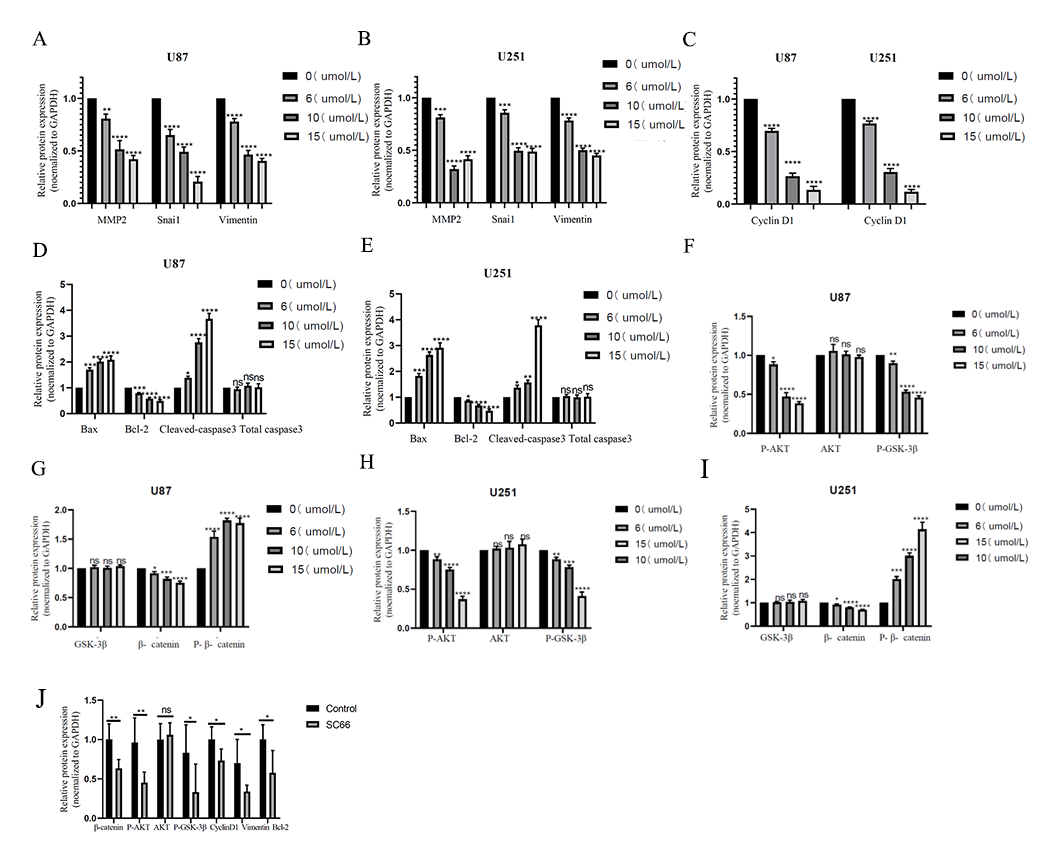

Supplement: Supplementary file 4 [file Image_1.tif]

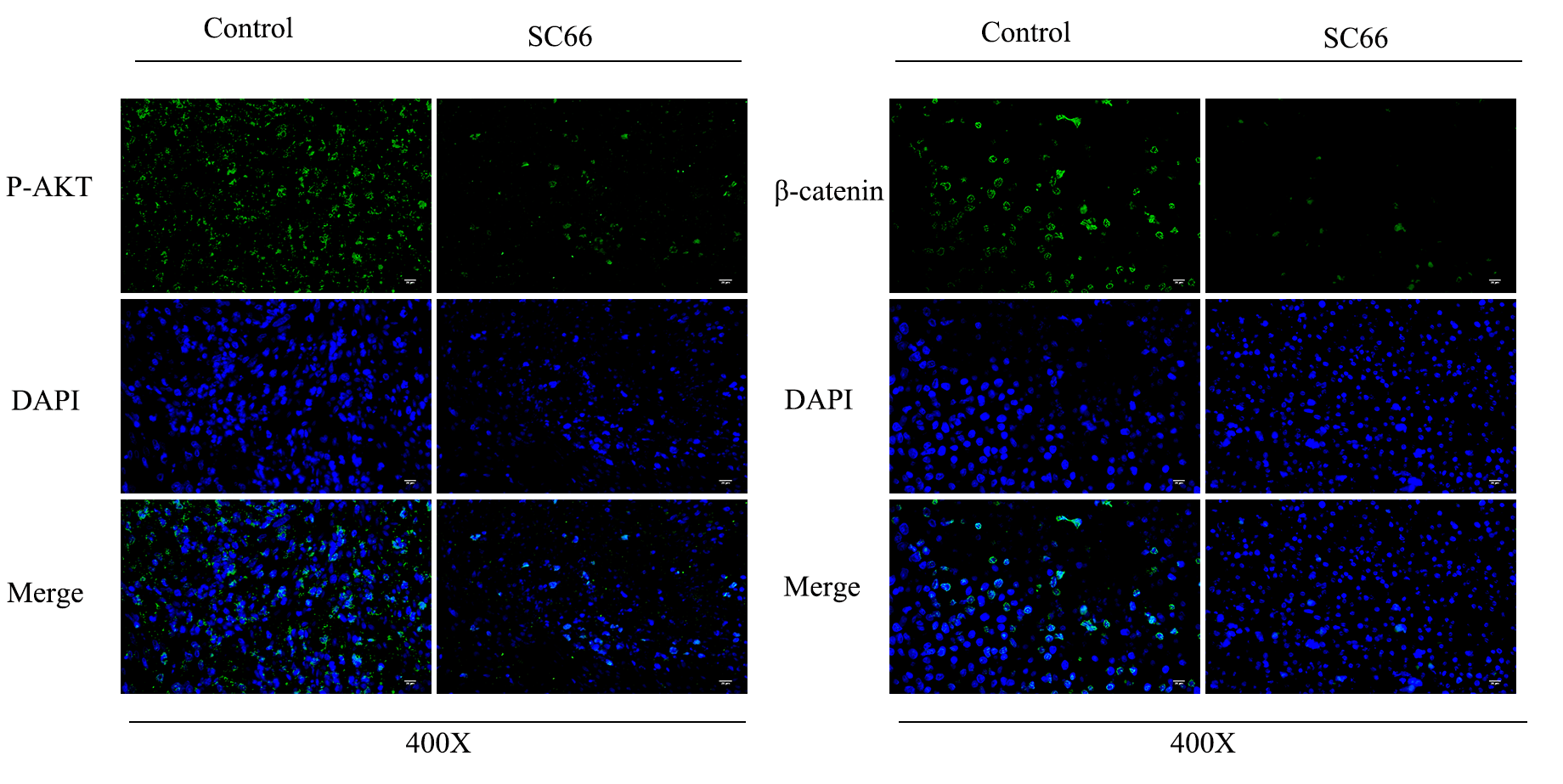

Supplement: Supplementary file 5 [file Image_2.tif]
